# Diffusion MRI sampling schemes bias diffusion metrics and tractography

**DOI:** 10.3389/fnimg.2026.1670604

**Published:** 2026-02-25

**Authors:** Ivanei Bramati, Diego Szczupak, Marina Carneiro Monteiro, Fernanda Meireles, Daniel Menezes Guimarães, Ryan J. Dean, Lynn K. Paul, Fernanda Tovar-Moll

**Affiliations:** 1 Department of Brain Connectivity and Plasticity, D’Or Institute for Research and Education, Rio de Janeiro, Brazil; 2 IRC5 – International Research Consortium for the Corpus Callosum and Cerebral Connectivity, Rio de Janeiro, Brazil; 3 Neurobiology Department, University of Pittsburgh, Pittsburgh, PA, United States; 4 Department of Neuroscience, Washington University in St Louis Medical School, St. Louis, MO, United States; 5 Division of Humanities and Social Sciences, California Institute of Technology, Pasadena, CA, United States

**Keywords:** anomalous white-matter bundles, corpus callosum dysgenesis, diffusion metrics, diffusion MRI, harmonization, tractography

## Abstract

**Introduction:**

Diffusion MRI is increasingly used to study white-matter architecture, but tractography and diffusion metrics can be biased by different sampling schemes. We assessed systematic differences across four common protocols—single-shell high-angular resolution diffusion imaging (HARDI), Siemens clinical multi-shell (Sms), diffusion spectrum imaging (DSI), and Human Connectome Project multi-shell (HCPms)—in healthy adults and individuals with corpus callosum dysgenesis (CCD).

**Methods:**

All data were acquired on a single 3 T scanner and processed uniformly to extract fractional anisotropy (FA), mean diffusivity (MD), effective contrast-to-noise ratio (eCNR), and orientation dispersion within the corpus callosum (CC), corona radiata (CR), and centrum semiovale (CSO). In controls, we measured tract volumes for CC, bilateral CR, anterior commissure (AC) and posterior commissure (PC), and streamline counts for AC and PC; in CCD, we quantified volumes of the Probst and sigmoid bundles.

**Results:**

Across participants, FA and MD showed moderate cross-scheme correlations for most ROIs, but matched means were rare (only Sms–HARDI in CC). eCNR and dispersion exhibited few cross-scheme correlations; however, means were similar for eCNR between Sms and HCPms and for dispersion among HARDI, DSI, and HCPms. Tract-based volumes correlated across Sms, DSI, and HCPms for CC in controls and for the right sigmoid and both Probst bundles in CCD. DSI and HCPms yielded similar volumes in all ROIs (controls and CCD). In controls, Sms volumes agreed with DSI/HCPms in CR, but were lower in CC and in all CCD ROIs. HARDI produced higher volumes in CC and bilateral CR in controls and in all CCD ROIs. For AC and PC in controls, tract-based means (volumes, streamlines, streamlines/volume) were consistent across schemes; nonetheless, correlations were limited—streamlines and streamlines/volume correlated for Sms, DSI, and HARDI in AC, and for DSI and HCPms in PC.

**Discussion:**

These findings demonstrate systematic differences in voxel-wise metrics and tractography outcomes from four diffusion-sampling schemes. In addition to qualitatively informing attempts to consolidate or contrast data across schemes, future work could explore regression-based harmonization—and other methods—to reduce residual bias and enable pooled analyses across diverse protocols.

## 1 Introduction

Diffusion-weighted magnetic resonance imaging (dMRI) exploits the random motion of water molecules to noninvasively probe tissue microstructure and infer fiber orientation (Baliyan et al., 2016; Le Bihan et al., 2001). By fitting mathematical models to these measurements, voxel-wise metrics—such as fractional anisotropy (FA) and mean diffusivity (MD)—can be derived, and tractography algorithms can reconstruct the trajectories of white-matter pathways (Mori and van Zijl, 2002). Yet dMRI outcomes are highly sensitive to how the diffusion-encoding space (q-space) is sampled, as well as hardware, acquisition settings, and reconstruction algorithms (Pinto et al., 2020). This sensitivity gives rise to inter- and intra-site variability that complicates data pooling across centers and in longitudinal studies (Vollmar et al., 2010).

Classical diffusion tensor imaging (DTI) acquires a single “shell” of relatively low-b diffusion gradients (*b* ≤ 1,000 s/mm^2^), in a modest number of directions (*n* ≤ 30), yielding rapid scans and high signal-to-noise on clinical scanners (Basser et al., 1994). However, because DTI assumes Gaussian diffusion, it fails in regions of complex fiber architecture—often producing misleading estimates of orientation and microstructural density, a phenomenon known as the “cross-fiber problem” (Jeurissen et al., 2013).

High angular resolution diffusion-weighted imaging (HARDI) addresses this by sampling a large number of diffusion gradient directions—typically over 50—at a single *b*-value (≥1,000 s/mm^2^), thereby improving sensitivity to fiber crossings and allowing accurate estimation of multiple orientations within each voxel (Tuch et al., 2002). Multi-shell acquisitions extend this approach by sampling multiple b-values, enabling the capture of non-Gaussian diffusion behavior that reflect hindered and restricted water motion within complex tissue microenvironments (Assaf and Pasternak, 2008; Henriques et al., 2021). These richer datasets underpin advanced diffusion models—such as the Composite Hindered and Restricted Model of Diffusion (CHARMED; Assaf and Basser, 2005) and Neurite Orientation Dispersion and Density Imaging (NODDI; Zhang et al., 2012)—that disentangle Gaussian from non-Gaussian components and yield more accurate microstructural measurements. At the extreme, diffusion spectrum imaging (DSI) which corresponds to the grid-based sampling scheme, densely samples a wide range of diffusion directions across multiple shells to reconstruct the full diffusion propagator, offering a comprehensive yet time-intensive map of tissue architecture (Wedeen et al., 2005).

As large-scale, multi-site dMRI studies become essential for investigating rare disorders and achieving robust statistical power, harmonizing diffusion protocols is critical. Differences in q-space sampling can introduce systematic biases in both voxel-level metrics and tractography outcomes (Jones and Cercignani, 2010; Teipel et al., 2011). Moreover, dMRI provides a suite of microstructural measures—most notable FA and MD—computed independently in each voxel to reflect fiber density, myelination, and fiber coherence (Assaf and Pasternak, 2008). These metrics form the basis for downstream connectivity analysis but, by themselves, do not capture the spatial trajectories that tractography reconstructs. Diffusion MRI tractography overcomes this limitation by propagating streamlines along voxel-wise principal diffusion directions, enabling three-dimensional reconstruction and quantitative analysis of white-matter pathways (Mori and van Zijl, 2002). This approach has become an essential tool for mapping brain connectivity in both research and clinical applications.

Tractography algorithms fall into two broad categories. Deterministic tractography follows the single most likely diffusion direction in each voxel to generate continuous streamlines (Conturo et al., 1999; Mori et al., 1999). Although computationally efficient, it tends to fail in regions of fiber complexity—where crossings, fanning, or sharp turns occur—yielding spurious terminations or false continuities (Jeurissen et al., 2013). Probabilistic tractography, by contrast, samples distribution of possible orientations at each voxel, producing potential pathways that better capture uncertainty in areas of complex anatomy (Behrens et al., 2007). While more robust to crossing fibers, this approach demands greater computational resources and can complicate the interpretation of connectivity estimates. Although voxel-wise diffusion metrics are calculated independently, tractography inherently depends on spatial continuity of fiber orientations across neighboring voxels. Consequently, errors or biases in estimating the orientation distribution functions (ODF) or tensor fields can propagate along streamlines (Jones, 2010), and different tractography algorithms may yield divergent reconstructions (Bastiani et al., 2012).

The primary goal of this study is to systematically assess how different dMRI acquisition schemes influence both voxel-wise diffusion metrics and tractography outputs in neurotypical individuals and in patients with Corpus Callosum Dysgenesis (CCD). Including participants with CCD allows us to assess each protocol’s robustness in highly altered white-matter neuroarchitectures. dMRI and tractography inherently struggle when the neuroanatomy deviates from typical patterns—conditions exemplified by CCD, where the absence or malformation of the corpus callosum (CC) gives rise to diverse neuroarchitectural reorganization and compensatory pathways (Siffredi et al., 2013). Corpus callosum dysgenesis (CCD) as an umbrella term for developmental malformations of the corpus callosum. CCD phenotypes include complete dysgenesis, also known as callosal agenesis (CA) and defined as absence of the corpus callosum; partial dysgenesis, which preserves a small callosal remnant; and callosal hypoplasia (HP), a thin but continuous corpus callosum. CCD can produce a wide spectrum of neurologic outcomes, reflecting its variable impact on interhemispheric connectivity (Lee et al., 2004). Among the most consistent hallmarks are the Probst bundles—aberrant longitudinal fibers that fail to cross the midline and instead run ipsilaterally alongside the interhemispheric fissure (Probst, 1901). The Probst bundles have been linked to the lack of proper axonal guidance during development, highlighting the impact of disrupted interhemispheric connectivity on brain organization (Paul et al., 2007).

Another common finding is the reorganization of the anterior commissure, a smaller midline-crossing white-matter tract. In individuals with CCD, this structure can be enlarged and may take on a compensatory role in facilitating interhemispheric communication (Tovar-Moll et al., 2007). Similarly, the sigmoid bundles—arc-shaped tracts connecting anterior and posterior regions within the contralateral hemisphere—are frequently observed. These tracts appear to serve as an alternative intrahemispheric pathway, reflecting the brain’s adaptive mechanisms in response to callosal dysgenesis (Hannay et al., 2009). These anomalous tracts—and their marked variability across CCD patients—underscore the importance of studying structural brain connectivity in this population. Such investigations provide critical insights on how developmental anomalies reshape white-matter and influence functional outcomes (Siffredi et al., 2021; Szczupak et al., 2021). Because CCD profoundly reorganizes commissural pathways, it challenges the accuracy of diffusion metrics and tractography algorithms.

This study aims to evaluate systematic differences in derived diffusion metrics across common diffusion-sampling-scheme archetypes (single-shell, multi-shell, grid-based). We scanned all participants—including neurotypical controls and individuals with CCD—on the same 3 T Siemens scanner using four diffusion-encoding schemes: single-shell HARDI, Siemens clinical multi-shell (Sms), DSI and HCP-style multi-shell (HCPms). By keeping preprocessing pipelines and reconstruction algorithms constant, we isolate the effect of q-space sampling on key metrics (FA, MD, dispersion, and eCNR) and tractography outputs (tract volumes and streamlines counts). Following comparison of diffusion metrics and tractography output in atlas-based regions from scans of neurotypical adults, we extend our exploration to assess how neuroarchitectural diversity impacts each protocol’s sensitivity. This examination of CCD’s anatomical alterations and their influence on inter-scheme differences highlight the critical role of diffusion schemes in studies of atypical brain anatomies. Unlike previous studies comparing different scanners or cohorts, our within-cohort, within-scanner design controls for all variables except diffusion sampling. By including CCD participants—whose Probst and sigmoid bundles reflect profound structural reorganization—we extend protocol evaluation into atypical neuroarchitectures, an area rarely addressed in the literature.

Our findings will provide critical insights for multicenter dMRI studies by identifying which schemes most reliably capture white-matter architecture and guiding the harmonization and standardization of diffusion protocols. Evaluating how different diffusion schemes influence both voxel-wise diffusion metrics and tractography outcomes is essential to the validity of neuroimaging studies. By identifying protocols that produce the most consistent and reliable results, we can make better acquisition choices—thereby improving the reproducibility and accuracy of dMRI analysis in both typical and atypical populations.

## 2 Materials and methods

### 2.1 Participants

**Figure 1:**
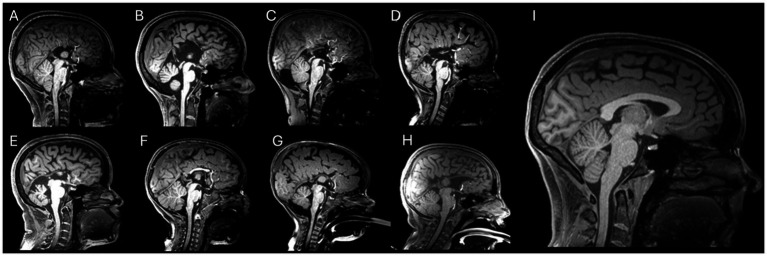
Mid-sagittal T1-weighted anatomy. CCD participants **(A–H)** and neurotypical control **(I)**. Subtypes: callosal agenesis **(A–E,G,H)**; callosal hypoplasia **(F)**. All images are central mid-sagittal slices displayed with identical field-of-view and window/level. This panel provides anatomical context for the within-subject comparisons across diffusion sampling schemes.

Twelve healthy adults (eight females; age range 24–78 years, mean = 34.7 ± 14.7), with no evidence of neurological disease. The CCD cohort comprised eight individuals (six females; age range 1–32 years, mean = 12.82; sd = 10.59). Exact ages are listed in Table 1. The CCD cohort included seven participants with callosal agenesis (CA) and one with callosal hypoplasia (HP); none had additional central nervous system malformations. Figure 1 shows central mid-sagittal T1-weighted anatomy for all CCD participants (Figures 1A–H) and one neurotypical control (1I), with subtypes indicated (callosal agenesis: Figures 1A–E,G,H; callosal hypoplasia: Figure 1F). All images are displayed with identical field-of-view and window/level and provide anatomical context for the within-subject comparisons across diffusion sampling schemes. Table 1 lists Subject ID, age (years), sex, the fixed acquisition order (HCPms → Sms → DSI → HARDI), and callosal subtype. An experienced neurologist from the IDOR (Institute D’Or for Research and Education) research team recorded the clinical histories of CCD patients. All participants (or their legal guardians) provided written informed consent. The study complied with the Declaration of Helsinki and was approved by the local Ethics Committee (Copa D’Or/Instituto D’Or de Pesquisa e Ensino, IDOR; n^0^ 4.456.129). All procedures were conducted in the IDOR by a multidisciplinary team.

**Table 1:** CCD cohort demographics, fixed acquisition order, and callosal subtype.

Subj ID	Age (years)	Sex	Acquisition order	CCD subtype
SUBJ001	14,6	F	HCPms, Sms, DSI, HARDI	CA
SUBJ002	24,0	F	HCPms, Sms, DSI, HARDI	CA
SUBJ003	8,3	M	HCPms, Sms, DSI, HARDI	CA
SUBJ004	8,2	M	HCPms, Sms, DSI, HARDI	CA
SUBJ005	32,4	F	HCPms, Sms, DSI, HARDI	CA
SUBJ006	3,4	F	HCPms, Sms, DSI, HARDI	HP
SUBJ007	1,4	F	HCPms, Sms, DSI, HARDI	CA
SUBJ008	10,0	F	HCPms, Sms, DSI, HARDI	CA

Columns: Subject ID; Age (years); Sex; Acquisition order (HCPms, Human Connectome Project multi-shell; Sms, Siemens multi-shell; DSI, diffusion spectrum imaging; HARDI, high angular resolution diffusion imaging); Callosal subtype (callosal agenesis—CA; callosal hypoplasia—HP).

### 2.2 dMRI acquisition

All scans were acquired on a single Siemens 3 T Prisma scanner (Siemens Healthcare, Erlangen, Germany) with a 32-channel head coil and high-performance gradients (80 mT/m, slew rate 200 mT/m/s), located at the D’Or Institute for Research and Education facility. A multiband echo planar imaging (EPI) sequence (MB = 4) from the Center for Magnetic Resonance Research (CMRR)^1^ was used for all dMRI acquisitions (Feinberg and Setsompop, 2013; Harms et al., 2018; Setsompop et al., 2012). Geometric parameters were held constant across protocols: isotropic voxel size = 1.5 × 1.5 × 1.5 mm^3^; field of view (FOV) = 210 mm; 92 axial slices; repetition time (TR) = 3,230 ms; echo time (TE) = 90 ms; phase-encoding anterior to posterior (AP); no in-plane acceleration. Participants were instructed to remain still. To minimize head motion during acquisition, foam padding and chin straps were secured over the participants’ foreheads. All diffusion acquisitions were collected in a fixed order for every participant: HCPms → Sms → DSI → HARDI. No randomization or repetition across sequences was used (Table 1). Before each acquisition, automatic system adjustments were performed to optimize magnetic field homogeneity and radio frequency (RF) transmission. The adjustment strategy was set to Standard, with B0 shim in *Standard* mode and B1 shim using the *TrueForm* algorithm. Adjustment tolerance was set to Auto, and frequency adjustment was enabled. The adjustment volume was positioned at the isocenter in transversal orientation, with no rotation (0.0°), and dimensions of 210 mm (anterior–posterior), 210 mm (right–left), and 138 mm (foot–head), ensuring consistent shimming and RF calibration within the imaging volume. These system adjustments required approximately ~2 min and constituted the interval between consecutive diffusion runs.

#### 2.2.1 HCP multi-shell (HCPms) protocol

This follows HCP-D/A (Human Connectome Project in Development and Aging) specifications (Harms et al., 2018): four dMRI runs, each with two shells: *b* = 1,500 s/mm^2^ (92 directions), *b* = 3,000 s/mm^2^ (93 directions). Total directions = 185. Each direction was acquired twice: AP (anterior to posterior) & PA (posterior to anterior). Total time = 22 min 36 s.

#### 2.2.2 Siemens multi-shell (Sms) protocol

This is a multi-shell version of Siemens’ standard diffusion protocol: two shells; *b* = 1,000 s/mm^2^ (30 directions); *b* = 3,000 s/mm^2^ (64 directions); two b0 images (AP & PA). Total time = 6 min 25 s.

#### 2.2.3 DSI protocol

This utilized a “grid-258” diffusion sampling scheme (Yeh, 2025^2^): 14 b-shells; max b = 3,000 s/mm^2^; *b*-value/direction pairs: 0/1, 190/6, 375/12, 562/8, 750/6, 937/24, 1,125/24, 1,500/12, 1,687/30, 1875/24, 2062/24, 2,250/8, 2,437/24, 2,625/48, 3,000/6; one reversed-phase b0 (PA). Total time = 24 min 13 s.

#### 2.2.4 HARDI protocol

This acquisition was based on Siemens’ standard diffusion protocol: one shell, b = 1,000 s/mm^2^ (256 directions); one b0 image. A reversed-phase b0 (posterior to anterior, PA) was acquired for distortion correction. Total time = 14 min 58 s.

The order of diffusion acquisitions was kept consistent across all participants: HCPms, Sms, DSI, and HARDI. No randomization across sequences was applied; the same order was maintained for every participant to ensure consistency in data acquisition. Importantly, no participant repeated any of these sequences within the protocol. The specific acquisition parameters, including *b*-values, number of gradient directions, number of b₀ images, acquisition times, phase-encoding schemes, and voxel sizes, are summarized in Table 2.

**Table 2:** Diffusion MRI acquisition parameters for the four sampling schemes.

Protocol	*b*-values (s/mm^2^)	Number of diffusion directions	Number of b0 images	Acquisition time	Phase encoding	Voxel size (mm^3^)	Features
HARDI	1,000	256	2 (AP & PA)	14′58”	AP	1.5	Single shell; high angular resolution
Sms	1,000, 3,000	30 (*b* = 1,000) 64 (*b* = 3,000)	2 (AP & PA)	6′25”	AP	1.5	Multi-shell; clinical Siemens protocol
DSI	0–3,000 (14 shells)	258	2 (AP & PA)	24′13”	AP	1.5	Grid-258 scheme; dense q-space sampling
HCPms	1,500, 3,000	2× (92 + 93) (AP and PA)	Multiple (AP & PA)	22′36”	AP & PA	1.5	Multi-shell; HCP Lifespan protocol

Listed are *b*-values, number of diffusion directions, number of b₀ images, acquisition time, phase-encoding, voxel size, and a brief description of each protocol’s features.

### 2.3 Imaging pre-processing

All data were converted to Brain Imaging Data Structure (BIDS) format (Gorgolewski et al., 2016, 2017) using heudiconv (v0.5.2-dev; Esteban et al., 2018). Quality control combined automated metrics and visual inspection. For each dMRI acquisition, we reviewed FSL eddy quality-control outputs (Bastiani et al., 2019), including absolute and relative displacement and the percentage of slice-wise outliers, and generated QUAD (QUality Assessment for DMRI; v1.0.2) and SQUAD (Study-wise QUality Assessment for DMRI) reports. QUAD was used for subject-level inspection of motion- and outlier-related QC metrics, whereas SQUAD aggregated QUAD outputs across participants and flagged subject-level datasets that behaved as outliers relative to the cohort. Accordingly, “acceptable ranges” were defined in a data-driven manner as QUAD-derived QC metrics within the cohort distributions and no SQUAD outlier flags. We also visually inspected QC plots/reports and representative diffusion-weighted volumes to identify artifacts (e.g., dropouts, ghosting, severe susceptibility distortions). No subject-level dataset was flagged as an outlier by SQUAD, and QUAD-derived QC metrics remained within acceptable ranges, although persistent dropout artifacts were detected in the same axial slices (orbitofrontal level) in the HARDI acquisition of one control participant. As the artifacts regions did not overlap with regions of interest of diffusion metrics estimates, no dataset was excluded from these analyses. However, the artifacts compromised the tractography reconstruction, and for this reason, the participant dataset was excluded from the tractography analyses. All dMRI processing was conducted with MRtrix 3.0.3 package (Tournier et al., 2019^3^) and FSL 6.0.4 toolbox (Smith et al., 2004^4^). For each AP and PA acquisition, a brain mask image was generated using dwi2mask command (Dhollander et al., 2016) which: firstly, averages all volumes of each separated b-value and finds an optimal threshold; and then, accordingly with the clean-scale parameter set, removes peninsula-like extensions by cutting bridges that connect them to the mask. After this, the maskfilter (Tournier et al., 2019) command was applied to the brain mask to dilate it.

### 2.4 dMRI processing pipeline

All dMRI datasets were first denoised (dwidenoise, MRtrix 3.0.3; Veraart et al., 2016) and corrected for Gibbs ringing artifacts (mrdegibbs, MRtrix 3.0.3; Kellner et al., 2016). We then applied dwifslpreproc, which is called FSL’s topup (FSL 6.0.4; Andersson et al., 2003) to correct susceptibility-induced distortions, and eddy (Andersson and Sotiropoulos, 2016) to correct eddy currents and subject motion. A B1 bias-field inhomogeneity correction was then applied (dwibiascorrect, MRtrix 3.0.3; Tustison et al., 2010). We used group quality reports from SQUAD to extract the contrast-to-noise ratio (CNR), which serves as a global index of diffusion MRI data quality. Because each diffusion protocol employed a different number of gradient directions per b-shell, we then calculated the effective CNR (eCNR) to normalize the standard CNR for these variations in angular sampling. In line with the HCP Lifespan definition (Harms et al., 2018), we derived the “raw” CNR for each b-shell b from FSL’s eddy. Using a Gaussian-process (GP) model (Andersson and Sotiropoulos, 2016), eddy defines raw CNR as the standard deviation of the GP predictions $\left(GP_{b}\right)$ divided by the standard deviation of the residuals $(\mathrm{re}s_{b}-$ the difference between the observations and the GP predictions) for that b-value shell:


$${\mathrm{CNR}}_{b}^{\left(\mathrm{raw}\right)}=\frac{\mathrm{std}({\mathrm{GP}}_{b})}{\mathrm{std}({\mathrm{res}}_{b})}.$$

We then computed the effective CNR by dividing the “raw” CNR per-image by the voxel volume and scaling it by the square root of the total number of volumes acquired at that b-value:


$${\text{eCNR}}_{b}=\frac{{\mathrm{CNR}}_{b}^{\left(\mathrm{raw}\right)}}{V_{\text{voxel}}}\;\sqrt{N_{b}}\;,$$

where $(V_{\text{voxel}})\;$ is the voxel volume (mm^3^) and $N_{b}$ is the number of volumes/directions in shell b . These normalizations follow the assumptions (explicit in Harms et al., 2018) that CNR increases approximately linearly with voxel volume but by the square root of the number of independent samples when averaging.

**Figure 2:**
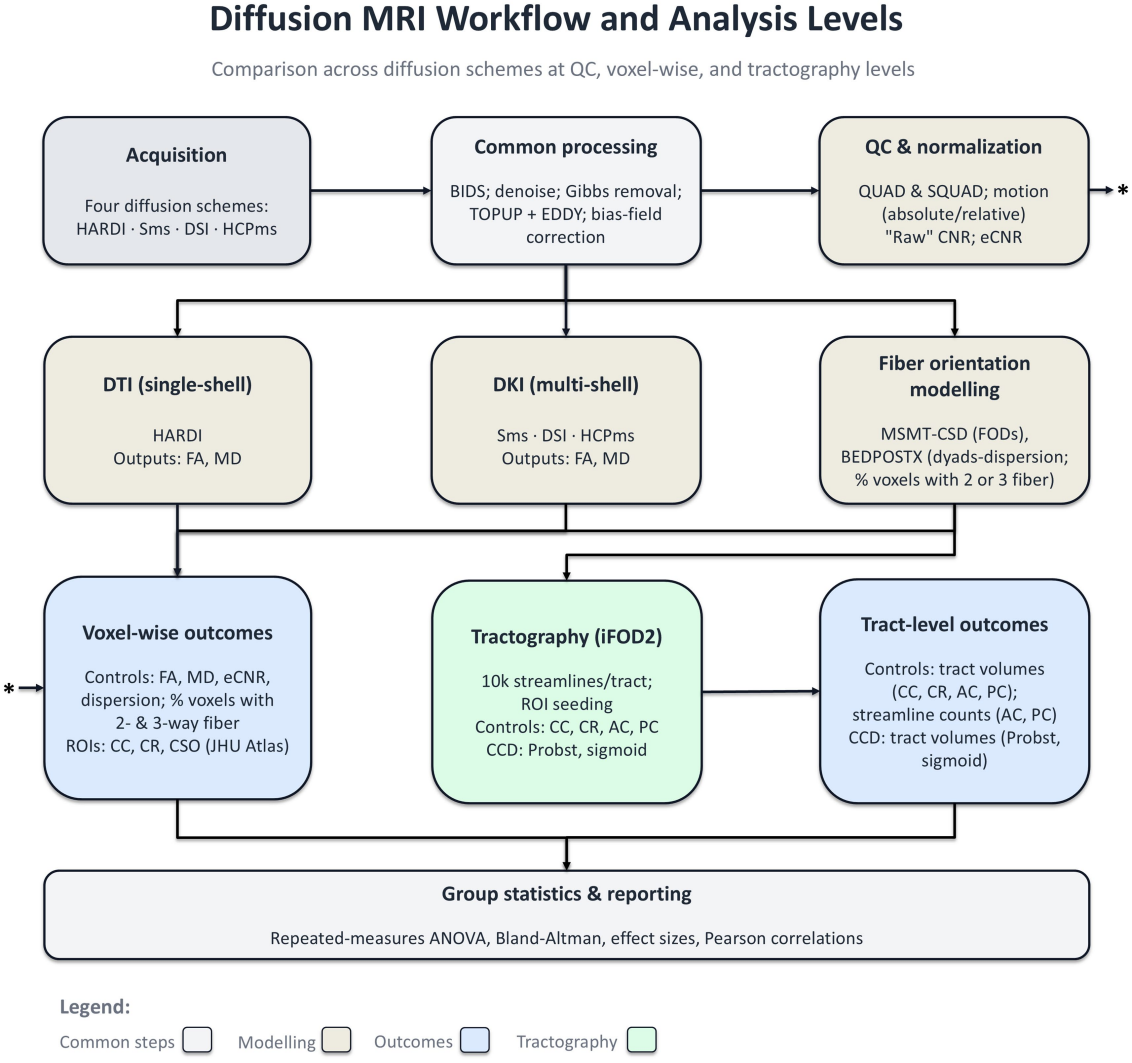
Diffusion MRI workflow, modeling, and analysis levels. Raw DWIs from four acquisition schemes (HARDI, Sms, DSI, and HCPms) undergo common preprocessing (BIDS conversion; denoising; Gibbs removal; TOPUP + EDDY; bias-field correction). QC & normalization compare diffusion schemes via absolute and relative motion, raw CNR and eCNR. Modeling then branches as follows: DTI (single-shell) → FA, MD (using tensor modelling); DKI (multi-shell) → FA, MD (using higher-order kurtosis modelling); Fiber-orientation modelling → MSMT-CSD FODs and BEDPOSTX multi-fiber estimates, yielding dispersion and the percentage of voxels with 2- or 3-way fiber crossings (CC/CR/CSO). Voxel-wise outcomes include FA, MD, eCNR, dispersion, and %*f*_2_ and %*f*_3_. Tract reconstruction uses probabilistic tractography (iFOD2) with 10 k streamlines per tract and ROI-based seeding. Tract-level outcomes include tract volumes for CC, bilateral CR, AC, and PC in controls and Probst/sigmoid bundles in CCD, plus streamline counts for AC and PC (controls). Group statistics summarize between-scheme differences (repeated-measures ANOVA, Pearson correlations, Bland–Altman). An asterisk (*) denotes a cross-link: the eCNR computed at the QC & normalization stage is also included among the voxel-wise outcomes (left blue panel). AC, anterior commissure; ANOVA, analysis of variance; BEDPOSTX, Bayesian estimation of diffusion parameters obtained using sampling techniques; BIDS, brain imaging data structure; CC, corpus callosum; CCD, corpus callosum dysgenesis; CNR, contrast-to-noise ratio; CR, corona radiata; CSO, centrum semiovale; DKI, diffusion kurtosis imaging; DSI, diffusion spectrum imaging; DTI, diffusion tensor imaging; eCNR, effective contrast-to-noise ratio; EDDY, eddy-current/motion correction; FA, fractional anisotropy; FODs, fiber orientation distributions; HARDI, high angular resolution diffusion imaging; HCPms, human connectome project multi-shell protocol; iFOD2, second-order integration over fiber orientation distributions; JHU atlas, Johns Hopkins University white-matter atlas; MD, mean diffusivity; MSMT-CSD, multi-shell, multi-tissue constrained spherical deconvolution; PC, posterior commissure; QC, quality control; ROI(s), region(s) of interest; Sms, Siemens multi-shell protocol; TOPUP, susceptibility distortion correction.

All multi-shell datasets (Sms, DSI, and HCPms) were processed with DIPY’s diffusion kurtosis imaging (DKI) model (Garyfallidis et al., 2014^5^) to produce fractional anisotropy (FA) and mean diffusivity (MD; mm^2^/s) maps, while the single-shell HARDI dataset was fit to DIPY’s standard diffusion tensor model. By using DKI on multi-shell data, we remove non-Gaussian bias at high b-values. Making FA and MD directly comparable across all schemes. In addition, we quantified the fiber-orientation uncertainty (dyads-dispersion) for every dataset using FSL’s BEDPOSTX which employs a Bayesian approach to model crossing fibers within each voxel (Behrens et al., 2007). To quantify the prevalence of crossing fibers across schemes, we ran FSL’s BEDPOSTX with up to three fiber populations *per* voxel, obtaining mean of the probabilistic distribution of the anisotropic volume fraction (mean *f*_1_, *f*_2_, and *f*_3_ samples). An overview of the diffusion processing and analysis workflow is provided in Figure 2.

Because nonlinear voxel-wise diffusion modeling is computationally intensive, all processing was executed on IDOR’s internally developed, cluster-based platform hosted on Amazon Web Services (AWS), enabling scalable parallel execution across compute nodes while maintaining robust data security and compliance.

### 2.5 ROI selection

**Figure 3:**
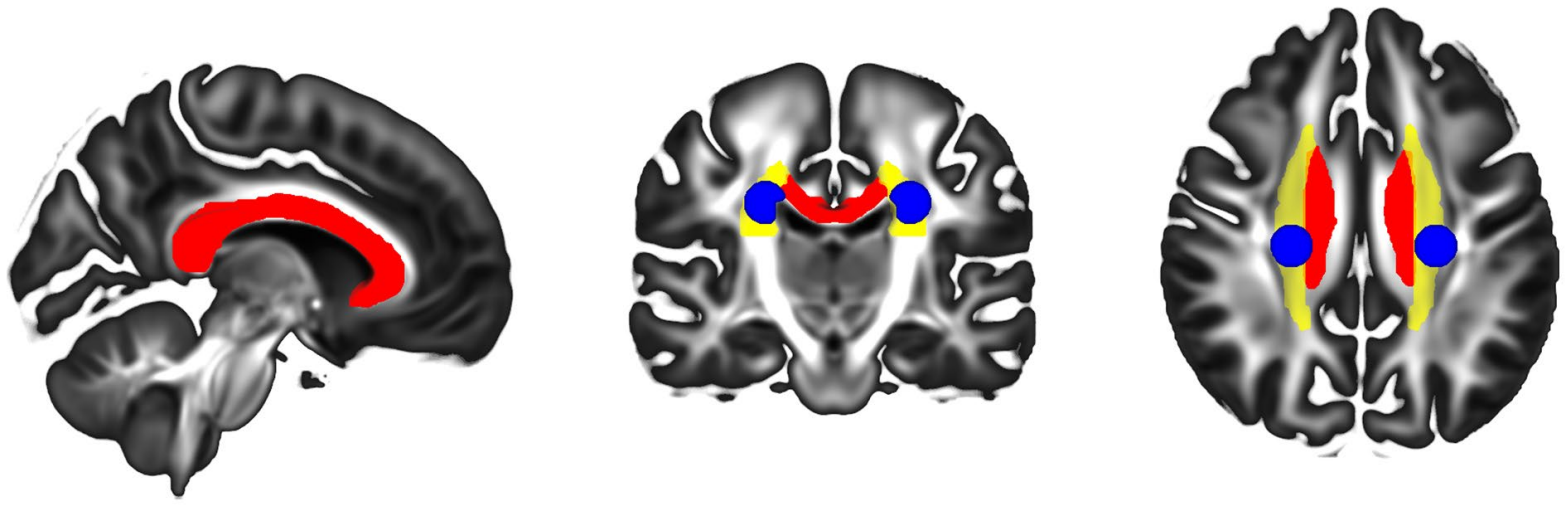
Regions of interest in control participants. Selected ROIs used to extract the diffusion metrics (FA, MD, and dispersion) and quality metrics (eCNR): corpus callosum (red), corona radiata (yellow), and centrum semiovale (blue). The corpus callosum and corona radiata were also used as seed masks for tractography.

Comparison of diffusion metrics was conducted in the control group using three bihemispheric white-matter ROIs—the corpus callosum (CC), the corona radiata (CR), and the centrum semiovale (CSO)—were defined using the JHU DTI atlas (Mori and Crain, 2005; Figure 3). We chose the CC and CR because they consist of coherently aligned fibers with few crossing fibers, supporting functions like interhemispheric integration, motor control, and higher-order functions (Goldstein et al., 2022). By contrast, the CSO contains interwoven fibers from the cingulum, CR, and CC, creating complex crossings that challenges both the acquisition of robust diffusion measurements and tractography (Avants et al., 2011). These atlas-defined ROIs were applied to the control group only; due to the marked anatomical variability in CCD (including absent or malformed corpus callosum), voxel-wise ROI metrics were not computed for the CCD cohort. For each ROI, diffusion metrics (FA and MD) were computed as the mean across all voxels within the ROI mask in native diffusion space; no additional voxel-wise outlier exclusion (e.g., interquartile-range trimming) was applied.

We also computed the percentage of voxels whose *f*_2_ (second fiber; 2-way) or *f*_3_ (third fiber; 3-way) exceeded a fixed threshold (0.05 and 0.10 within each ROI mask Behrens et al., 2007). As implemented, thresholded maps (mean_f2samples or mean_f3samples) were masked by the ROI, and voxel counts were obtained with fslstats -V; percentages were expressed relative to the ROI voxel. This was implemented using FSL (fslmaths, fslstats) handled by an in-house Python script.

### 2.6 Tractography

In the control group, tractography was carried out to reconstruct three tracts: CC, left CR, and right CR. For the CC, two parasagittal ROIs were manually delineated on the CC and used as inclusion ROIs (Supplementary Figure S1A). To reconstruct the corticospinal tract, one inclusion ROI was placed in the corona radiata direct beneath the primary motor cortex and a second one, in the lower brainstem, at the level of the cerebral peduncles (Supplementary Figure S2A). In CCD patients, Probst and sigmoid bundles were reconstructed using two large coronal-plane ROIs: one encompassing the cingulate bundle bilaterally and another at the level of the splenium, surrounding the longitudinal medial bundles. Probst bundles were tracked with ipsilateral ROI pairs (Supplementary Figure S3A), whereas sigmoid bundles used contralateral ROI combinations (Supplementary Figure S4A). ROIs placement was performed in each subject’s native DWI space and was guided by FA-color-coded maps and Fiber Orientation Distribution (FOD) images assisted by co-registered high-resolution T1-weighted images, ensuring accurate correspondence between diffusion information and anatomical landmarks. All ROI delineations were conducted by a single operator with formal training and experience in neuroanatomy and diffusion MRI tractography to ensure consistency across subjects. Tract volumes were computed from binary masks in FSL and streamlines counts—including those for the anterior commissure (AC) and posterior commissure (PC)—were extracted to compare reconstruction consistency across the four diffusion schemes.

Tractography was performed in MRTrix 3.0.3 for all subjects and diffusion schemes. White-matter, gray-matter, and cerebrospinal fluid response functions were estimated with the unsupervised Dhollander algorithm dwi2response. Fiber orientation distributions (FODs) were then computed using the multi-shell multi-tissue constrained spherical deconvolution (MSMT-CSD) algorithm (Jeurissen et al., 2014; Tournier et al., 2004). For single-shell acquisitions, MSMT-CSD was applied using a single-tissue approach, generating response functions from the available data to estimate FODs while accounting for partial volume effects.

Streamlines were generated (tckgen) using the second-order Integration over Fiber Orientation Distributions (iFOD2) method (Tournier, 2010). For control participants, we set up to 10,000 streamlines per target and seeded CC ROIs from the right (seed region) to the left (inclusion region). For both left and right CR, we seeded the ROIs from the lower brainstem (seed region) to the superior CR (inclusion region). We used 10,000 streamlines per tract to balance sampling and computational cost, in line with prior evidence for convergence at modest sampling (5,000 samples/seed) and common tractometry practice (Besseling et al., 2012; Meisler and Gabrieli, 2022; Toselli et al., 2017). This limit provides a reasonable balance between computational feasibility and sampling of the probability distribution. The algorithm accepted only streamlines that traversed the inclusion ROI. A FOD amplitude of 0.06 was used as the termination threshold, with a 250 mm maximum length for any tract. Voxels were included in the tract-volume mask if traversed by at least one streamline (minimum threshold: 1 streamline per voxel). The streamline-to-volume ratio was defined as the total number of streamlines reconstructed for a given tract divided by the tract volume. Tract volume was computed as the number of voxels included in the tract mask (derived from the tractogram) multiplied by the voxel size (1.5 × 1.5 × 1.5 mm). This ratio provides a normalized index of tract density that accounts for differences in tract size.

To investigate the unexpected reconstruction of anomalous bundles in controls, we implemented, using the same algorithm, a virtual callosotomy, i.e., a computationally extraction of corpus callosum to simulate a complete interhemispheric disconnection of callosal streamlines (Vakharia et al., 2020). Two seed regions were placed in the left and right occipital superior gyri, as defined by the Automated Anatomical Labelling atlas (AAL; Rolls et al., 2020) and registered to diffusion space with antsRegistrationSyNQuick (ANTS). To avoid false positive through the splenium of the corpus callosum—which normally connects interhemispheric visual areas—we manually defined an exclusion ROI within the CC. This ensured that only the atypical fiber pathways characteristic of callosal dysgenesis were captured.

For CCD participants, we generated up to 10,000 streamlines per target in each hemisphere, using two seed regions. For the Probst bundle, seeds were manually placed at the anterior and posterior cross-sections of the bundle, at roughly its midpoint on a coronal T1 image. The sigmoid bundle was tracked using the same ROIs, but by combining the frontal ROI of one hemisphere with the posterior ROI of the opposite hemisphere. To accommodate the highly variable anatomy in CCD, all ROI placements were individualized (not atlas-based) rather than tied to a fixed anatomical plane.

**Figure 4:**
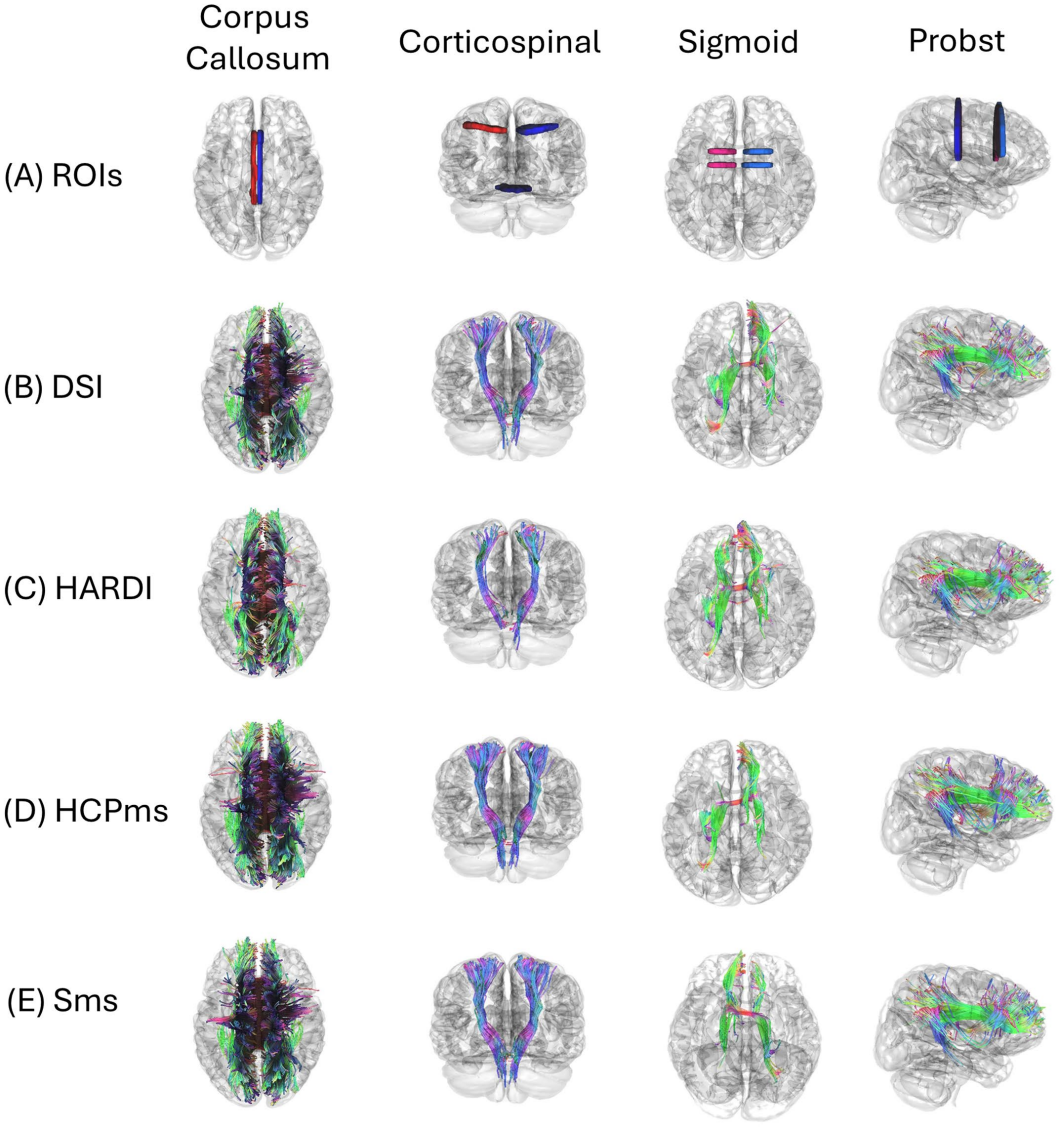
Tractography across diffusion sampling schemes in a representative control and CCD participant. Bilateral corpus callosum ROIs—left (red) and right (blue)—are shown in **(A)**; reconstructions of white-matter bundles are displayed across coronal, axial, and sagittal views, overlayed on a 3D glass-brain. **(B–E)** compare four diffusion schemes, using identical seeding and tracking parameters: **(B)** DSI (diffusion spectrum imaging), **(C)** HARDI (high-angular resolution diffusion imaging), **(D)** HCPms (Human Connectome Project multi-shell), and **(E)** Sms (Siemens multi-shell). Streamlines are RGB-encoded by orientation (red: left—right; green: anterior—posterior; blue: superior—inferior). This figure summarizes the tract projections detailed in Supplementary Figures S1–S4; see Supplementary Material for full 3D renderings and additional views.

To improve and standardize visualization, we imported the tractography outputs into DSI Studio, an open-source tool for diffusion MRI analysis and visualization (Tran and Shi, 2015). Figure 4 provides a concise overview of tractography reconstructions ROIs (3A) across the four diffusion schemes (DSI (3B), HARDI (3C), HCPms (3D), Sms (3E)) using identical seeding and tracking settings, with coronal, axial, and sagittal views overlaid on a 3D glass-brain for a representative control and a CCD participant. Full 3D renderings and additional views remain in Supplementary Figures S1–S4. We used a control subject to illustrate the CC (Supplementary Figure S1) and the corticospinal tract (Supplementary Figure S2), and a representative CCD case for the sigmoid bundle (Supplementary Figure S3) and Probst bundle (Supplementary Figure S4). Each tract was reconstructed independently under all four schemes to enable within-subject, across-scheme comparisons of typical and atypical neuroarchitectures.

### 2.7 Analysis

Results acquired under four diffusion schemes were compared using Pearson correlation to assess linear relationships, one-way repeated measures ANOVA with Tukey post-hoc comparisons of means across schemes, and Bland–Altman (BA) plots to evaluate agreement and detect any systematic bias between schemes (Bland and Altman, 1986, 1987). To account for the potential impact of participant motion on diffusion metrics, we also examined correlations between motion and diffusion metrics across schemes. In some CCD patients, sedation was used during imaging, making motion metric analysis less informative. Thus, motion evaluation was limited to the healthy control group.

In control participants, we compared the following outcomes generated by the four diffusion schemes: whole-brain metrics of absolute and relative motion, and diffusion metrics (FA, MD, eCNR, dispersion, percentage of voxels whose anisotropic volume fraction exceeds fixed thresholds) from 3 atlas-based regions of interest (CC, CR, and CSO). We also compared tractography-based outcomes from the four schemes: volume metrics in the CC, CR within each hemisphere, anterior commissure (AC), and posterior commissure (PC), as well as streamline counts in the AC and PC.

For CCD participants, atlas ROIs were not applied; tractography targeted the Probst and sigmoid bundles, each tract was reconstructed independently under all four schemes, and comparisons were limited to within-subject metrics.

For comparison of the four diffusion schemes one-way repeated measures ANOVA with Tukey post-hoc comparisons, boxplots, and Bland–Altman plots were generated using GraphPad Prism version 9.5.1^6^ and BA-plotteR (Goedhart and Rishniw, 2021). For each ROI (CC, CR, and CSO) and each diffusion scheme (HARDI, Sms, DSI, and HCPms), boxplots show the percentage of voxels whose anisotropic volume fraction exceeds fixed thresholds (0.05 and 0.1, the former being more permissive). Inter-scheme differences were assessed via One-Way ANOVA followed by a Tukey post-test. Boxplots follow Tukey’s convention (median line, inter-quartile range box, whiskers to 1.5 × inter-quartile range), with individual data points overlaid to show participant-level variability.

BA plots display each participant’s averaged value obtained from two given methods on the x-axis versus their difference on the y-axis, overlaid with the mean bias and 95% limits of agreement (with 95% confidence intervals around those limits generated in BA-plotteR; Goedhart and Rishniw, 2021). Differences failing the Shapiro–Wilk normality test were converted to percentages (difference/average), retested, and—if normally distributed and without evidence of proportional bias (i.e., the 95% CI of slope includes zero)—percentage was plotted in standard BA form using GraphPad Prism (version 9.5.1). However, if percentage BA plot was not normally distributed and/or indicated proportional bias, BA regression plots were generated in BA-plotteR.

## 3 Results

### 3.1 Motion metrics

**Figure 5:**
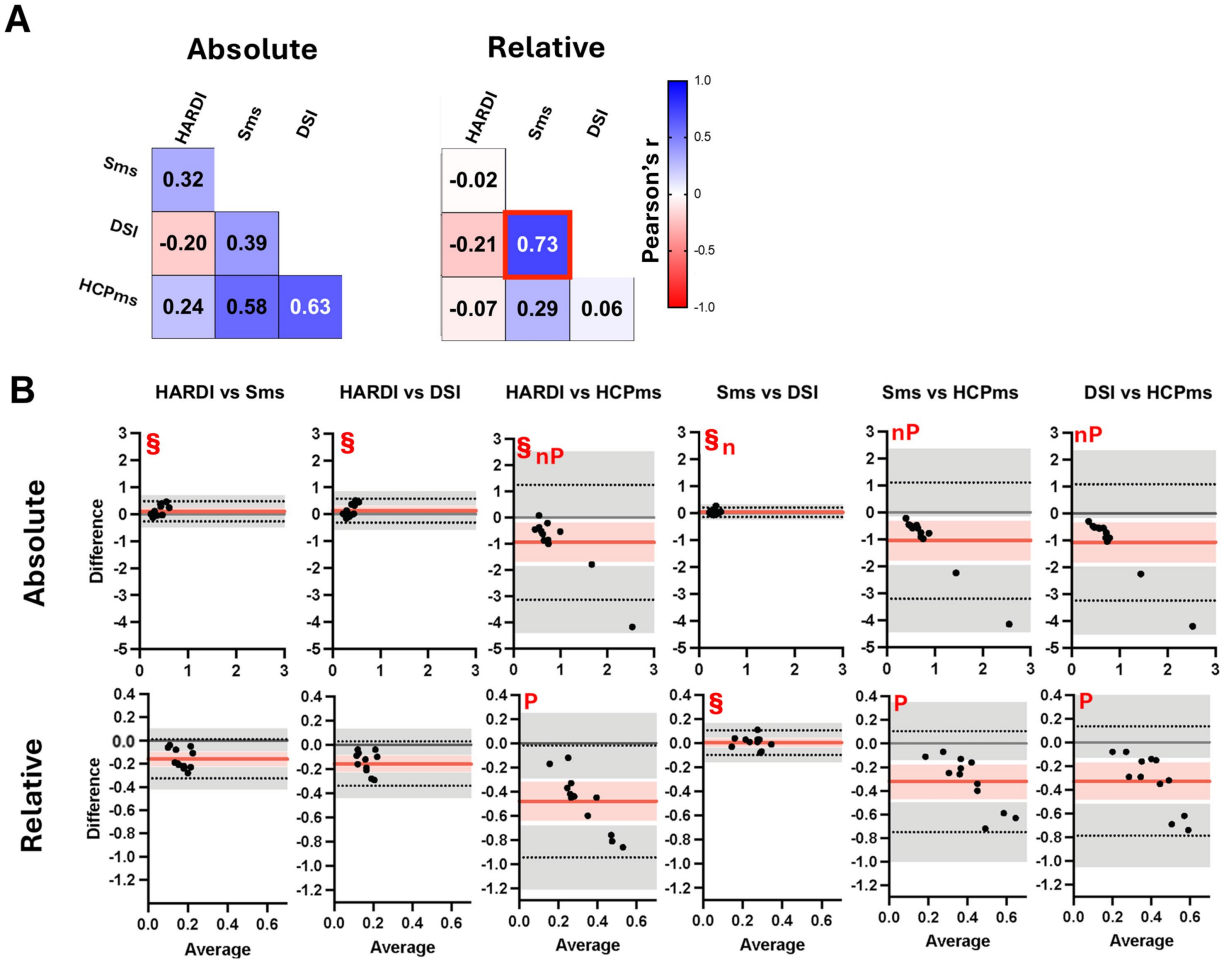
Motion metrics from 4 schemes. Plots show pairwise comparison of absolute and relative motion metrics from 4 schemes. **(A)** Pearson correlation *r* values. **(B)** Bland–Altman plots. Black dots represent individual participant data (average value from the two methods on the x-axis versus difference between values from the two methods on the y-axis). Horizontal red lines show the mean difference, with 95% confidence interval in pink. Dotted horizontal lines show limits of agreement with precision estimates (95% confidence interval) in grey. The equality value (0) is shown by a horizontal gray line. Subplots are scaled consistently across motion metric. Red box, correlation significant at Bonferroni-adjusted *p* < 0.05; ^§^ mean difference not significant at Tukey-adjusted *p* < 0.05; n, raw differences not normally distributed, but percent normally distributed, *p*, slope of the difference indicates proportional bias for raw scores, but not for percentage, *P*, proportional bias is evident for both raw scores and percent.

We assessed absolute and relative motion metrics from the dMRI data acquired under the four diffusion schemes to rule out participant movement as a source of bias. Although motion is generally assumed to be random, we examined correlations in motion metrics to see whether individual participants tended to move consistently across different scanning protocols. Establishing these patterns was essential to ensure that any observed differences in diffusion properties and tractography outcomes truly reflected the diffusion sampling schemes rather than variability in subject motion. The only significant relationship we observed was between the relative motion in the Sms and DSI acquisitions (Figure 5A), indicating that participants who moved more during Sms also tended to move again during DSI.

Repeated-measures ANOVA revealed significant variability on average for both absolute motion (*R*^2^ = 0.4769) and relative motion (*R*^2^ = 0.7516; Supplementary Table S1). Exploration of Tukey *post-hoc* comparisons between schemes suggests scan motion was not influenced by scan duration as Sms (the shortest acquisition time) and DSI (the longest) did not differ significantly for either absolute motion (mean difference = 0.028, 95% CI [−0.050, 0.107]) or relative motion (mean difference = −0.003, 95% CI [−0.042, 0.049]; Figure 5B), even after correcting for non-normality in absolute motion differences (Supplementary Figure S5). Notably, however, Figure 5B also reveals a systematic bias in the absolute motion values reported by the HCPms acquisition. For all comparisons, the mean difference is shifted in the negative direction, indicating that HCPms motion estimates tend to be higher than those from the other protocols (HARDI vs. HCPms, mean difference = −0.940, 95% CI [−1.912, 0.032]; Sms vs. HCPms, mean difference = −1.045, 95% CI [−2.001–0.089]; DSI vs. HCPms mean difference = −1.073, 95% CI [−2.033, −0.114]). This trend becomes increasingly pronounced for participants with more extreme motion values, producing a negatively skewed distribution that resembles an inverse correlation. Additional analyses of individual movement patterns and their impact on diffusion metrics are provided in Supplementary Figures S5, S6.

### 3.2 Diffusion metrics

**Figure 6:**
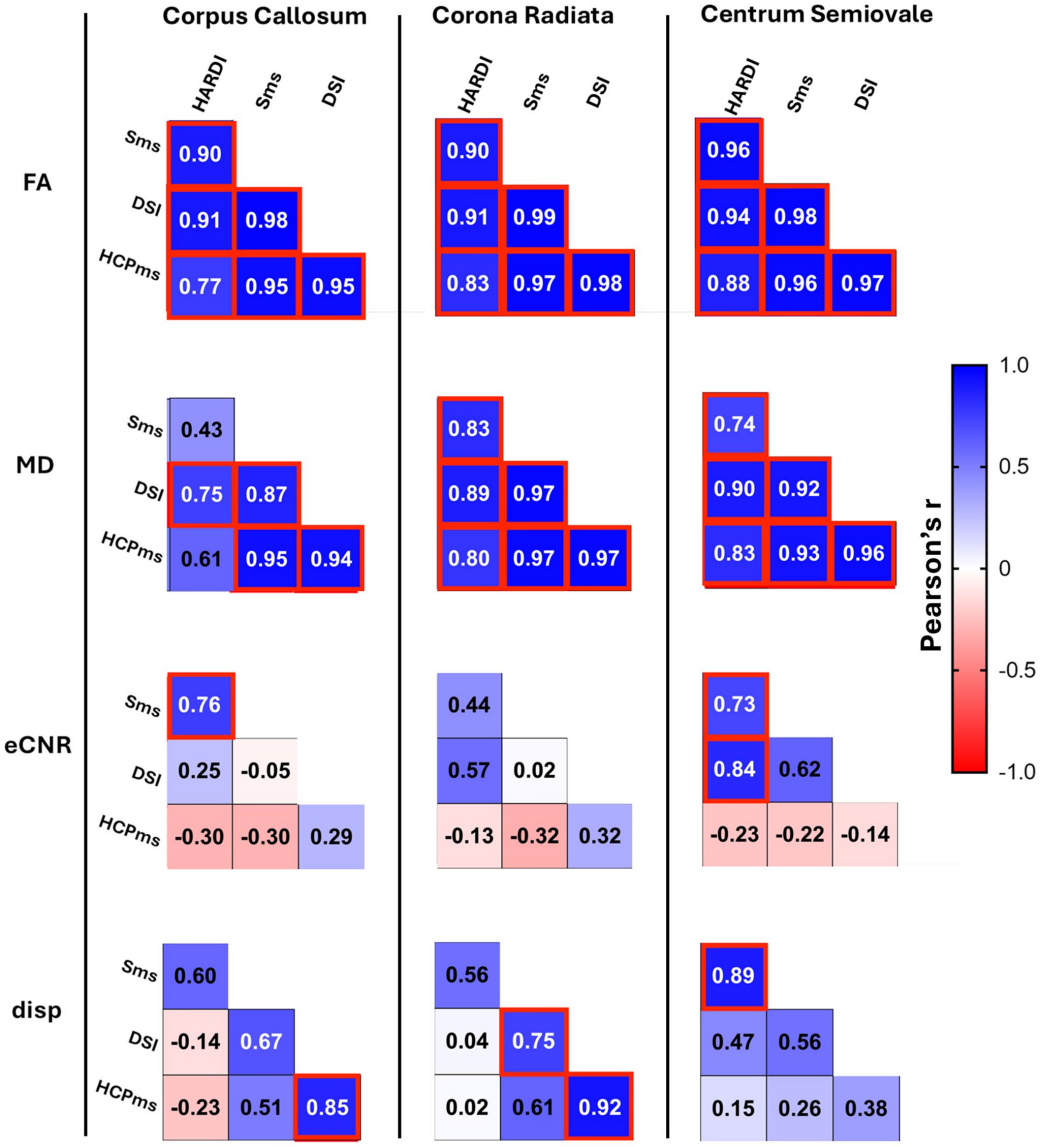
Correlation of diffusion metrics from four schemes for three regions of interest. Pair-wise Pearson correlations comparing diffusion metrics from four schemes. FA, fractional anisotropy; MD, mean diffusivity; eCNR, effective contrast to noise ratio; disp, dispersion; red box, correlation significant at Bonferroni-adjusted *p* < 0.05.

**Figure 7:**
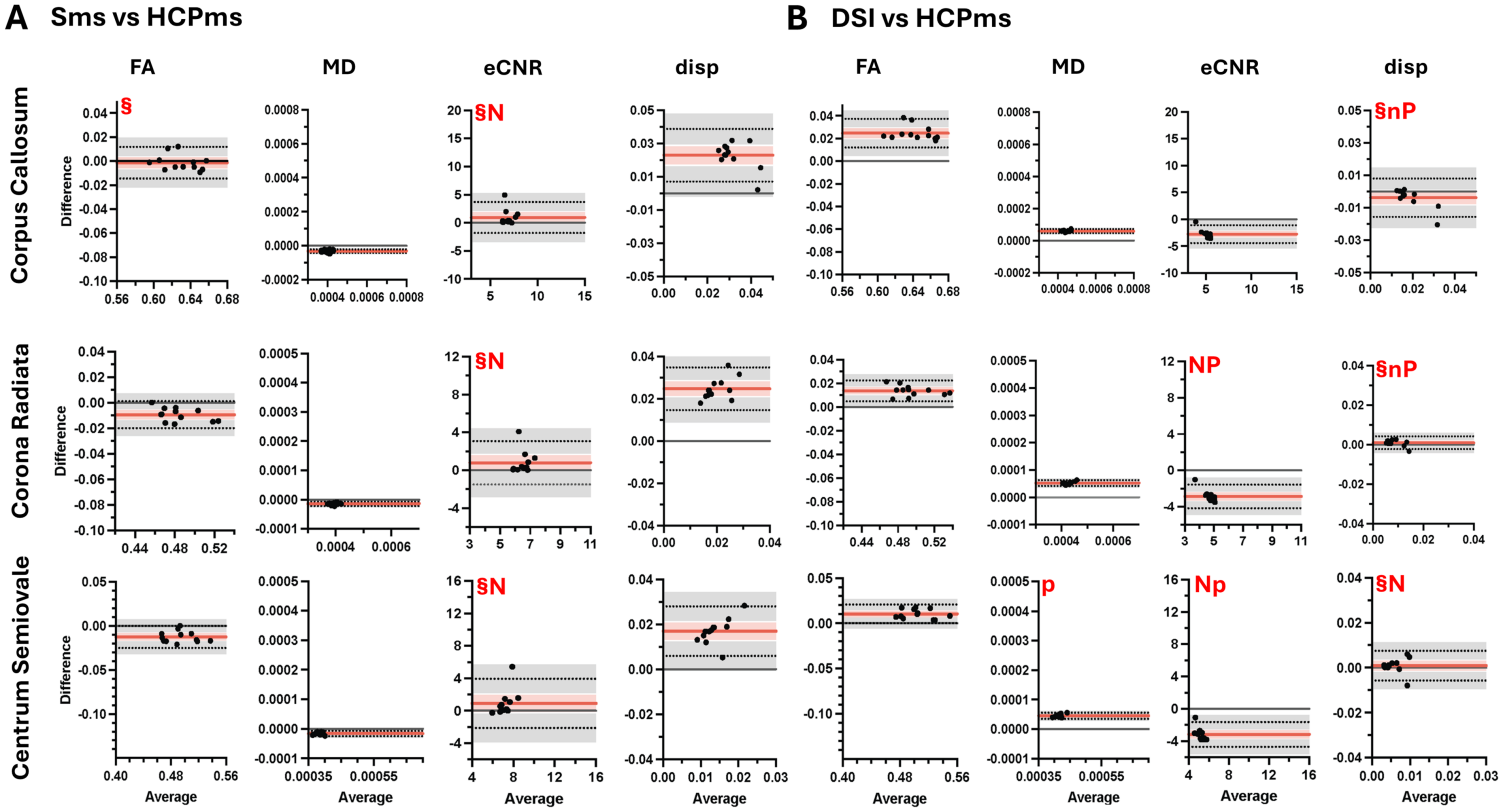
Bland–Altman plots comparing diffusivity metrics from diffusion schemes for three regions of interest. Plots show comparisons of diffusion metrics from SMS vs. HCPms **(A)** and DSI vs. HCPms **(B)**. FA, fractional anisotropy; MD, mean diffusivity; eCNR, effective contrast to noise ratio; disp, dispersion; ^§^mean difference not significant at Tukey-adjusted *p* < 0.05; n, raw differences not normally distributed, but percent normally distributed; N, raw differences and percent not normally distributed; p, slope of the difference indicates proportional bias for raw scores, but not for percent; P, proportional bias is evident for both raw scores and percent.

**Figure 8:**
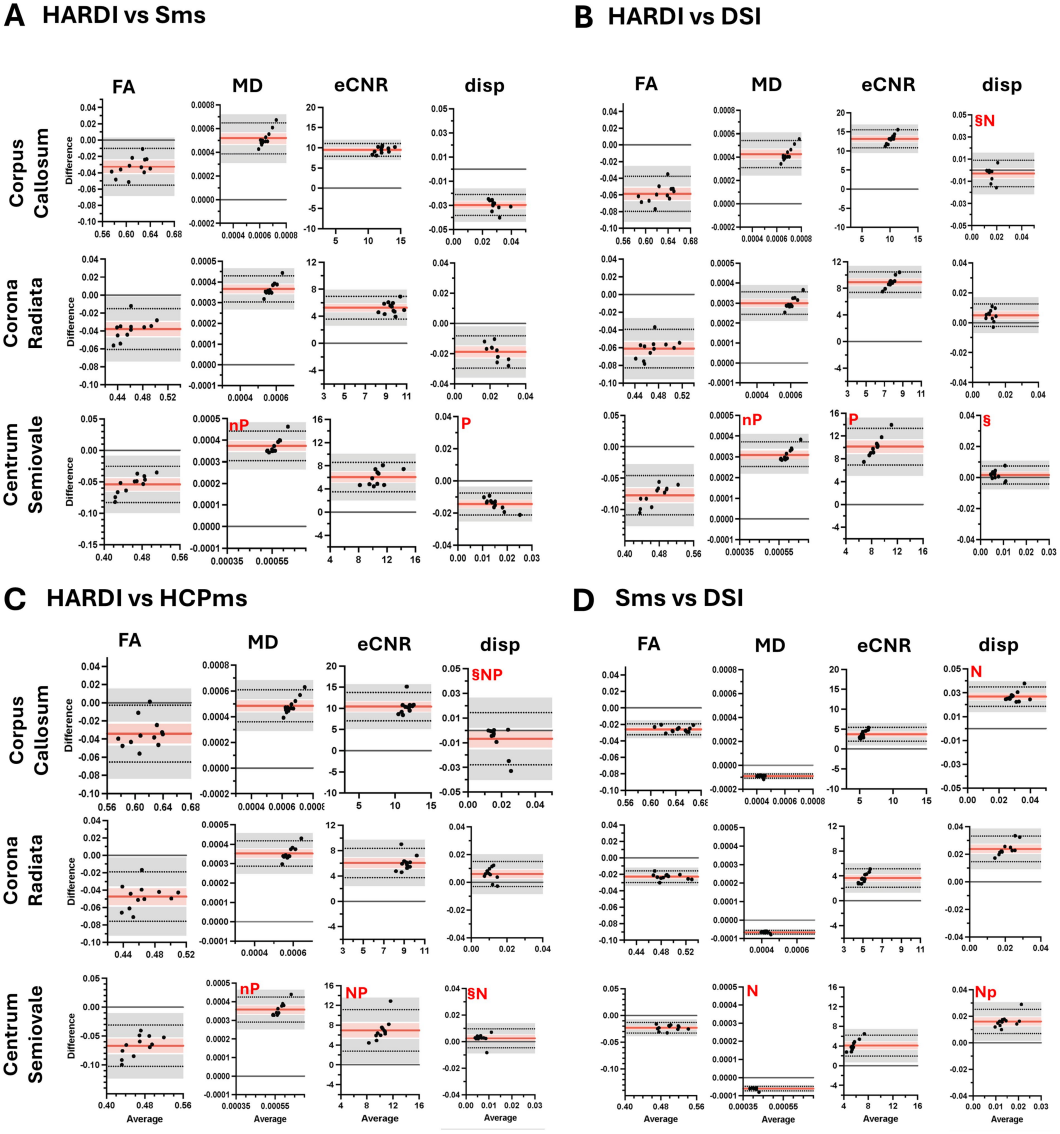
Bland–Altman plots comparing diffusivity metrics from schemes for three regions of interest. Plots show comparisons of diffusion metrics from HARDI vs. SMS **(A)**, HARDI vs. DSI **(B)**, HARDI vs. HCPMS **(C)**, SMS vs. DSI **(D)**. Black dots represent individual participant data (average value from the two methods on the x-axis versus difference between values from the two methods on the y-axis). Horizontal red lines show the mean difference, with 95% confidence interval in pink. Dotted horizontal lines show limits of agreement with precision estimates, with 95% confidence interval in grey. The equality value (0) is shown by a horizontal black line. Subplots of diffusion metric by region of interest are scaled consistently across comparisons **(A–D)**. FA, fractional anisotropy; MD, mean diffusivity; eCNR, effective contrast to noise ratio; disp, dispersion; ^§^mean difference not significant at Tukey-adjusted *p* < 0.05; n, raw differences not normally distributed, but percent normally distributed; N, raw differences and percent not normally distributed; p, slope of the difference indicates proportional bias for raw scores, but not for percent; P, proportional bias is evident for both raw scores and percent.

FA and MD values across all four diffusion schemes were highly correlated within each ROI, except for MD correlations in the CC for HARDI vs. Sms and HARDI vs. HCPms (Figure 6). However, 4-group ANOVAs (see Supplementary Table S1) found significant variability on average for both FA (R^2^ = 0.9264–0.9450) and MD (*R*^2^ = 0.9842–0.9922) and BA analysis showed that only Sms and HCPms yielded similar FA values in the CC (mean difference = −0.0014, 95% CI [−0.007, 0.004]; Figure 7A). All other two scheme comparisons of FA and MD exhibited systematic bias: HARDI produced lower FA and higher MD relative to the other three schemes (Figures 8A–C), while DSI yielded higher FA and MD than both Sms (Figure 8D) and HCPms (Figure 7B). Unlike FA, MD resulted in several comparisons which violated normality and / or revealed proportional bias as described in Supplementary Figure S7. Follow-up regression analyses of these anomalies indicate increased average MD is associated with increased deviation from HARDI (Supplementary Figures S7A–C) and increased variability in differences between Sms and DSI (Supplementary Figure S7D).

In contrast to FA and MD, eCNR showed few significant correlations between schemes (HARDI vs. Sms in CC and CSO, HARDI vs. DSI in CSO; Figure 6). However, as seen with FA, only Sms and HCPms yielded similar eCNR means (Figure 7A; CC mean difference = 0.934, 95% CI [−0.285, 2.154], CR mean difference = 0.786, 95% CI [−0.228, 1.800], CSO mean difference = 0.909, 95% CI [−0.435, 2.252]). Overall, BA plots confirmed a bias toward higher eCNR values for HARDI compared to all other schemes (Figures 8A–C) and lower eCNR values for DSI relative to HCPms and Sms (Figures 7B, 8D, respectively). As described in Supplementary Figure S8, non-normality and proportional bias were more prevalent in CSO and in HCPms comparisons, with ROI-specific patterns evident in the comparison of Sms and HCPms.

Although there were only a few significant correlations for dispersion values (DSI vs. HCPms in CC and CR, Sms vs. DSI in CR, Sms vs. HARDI in CSO; Figure 6), three schemes (HARDI, DSI, and HCPms) produced statically similar dispersion values in CC and CSO (HARDI vs. DSI CC mean difference = − 0.003, 95%CI [−0.008, 0.002], CSO mean difference = 0.002, 95%CI [−0.001, 0.004], Figure 8B; HARDI vs. HCPms CC mean difference = − 0.009, 95%CI [−0.016, 0.003], CSO mean difference = 0.002, 95%CI [−0.0007, 0.0056], Figure 8C; DSI vs. HCPms CC mean difference = − 0.004, 95%CI [−0.009, 0.001], CSO mean difference = 0.004, 95%CI [−0.002, 0.004], Figure 7B). DSI vs. HCPms also produced similar values in CR (mean difference = 0.0009, 95%CI [−0.0005, 0.0024], Figure 7B). In contrast, BA plots confirmed a bias toward higher Sms values than all other schemes (Figures 7A, 8A,D).

BA analysis showed that only one of the significant agreements of mean dispersion (HARDI vs. DSI in CSO) was not subject to proportional bias and/or non-normality (Supplementary Figure S9A). As detailed in Supplementary Figure S9, for all the comparisons in which non-normality and/or proportional bias remained after conversion to percent difference, BA regression plots revealed a positive association between average dispersion and variability of differences (Supplementary Figures S9B–I). Additionally, comparisons characterized by proportional bias in raw scores exhibited a negative association between average dispersion value and the average difference (Supplementary Figures S9C,F,G,H). Supplementary Table S5 displays the mean values for FA and MD for the controls.

Using BEDPOSTX with up to three fiber populations per voxel, we quantified, within each ROI, the percentage of voxels supporting a second (*f*_2_; 2-way) and a third (*f*_3_; 3-way) fiber population. As summarized in Supplementary Figure S10, the fraction of 2-way fiber voxels (thresholds *f*_2_ > 0.05 and *f*_2_ > 0.1) differed across schemes in CC, CR, and CSO. In the CC, Sms showed a lower number of 2-way fiber voxels when compared to all three other schemes. The differences were attenuated for 3-way fiber voxels at both thresholds, consistent with the high anisotropy of callosal tissue. In contrast, both CR and CSO showed robust scheme-dependent differences for 2- and 3-way fiber voxels, and these patterns persisted at both thresholds (including the more permissive *f*_3_ > 0.05), except for the CSO 2-way at 0.05 threshold where only Sms showed significantly lower number of voxels.

### 3.3 Tractography-based metrics

#### 3.3.1 Volumes

**Figure 9:**
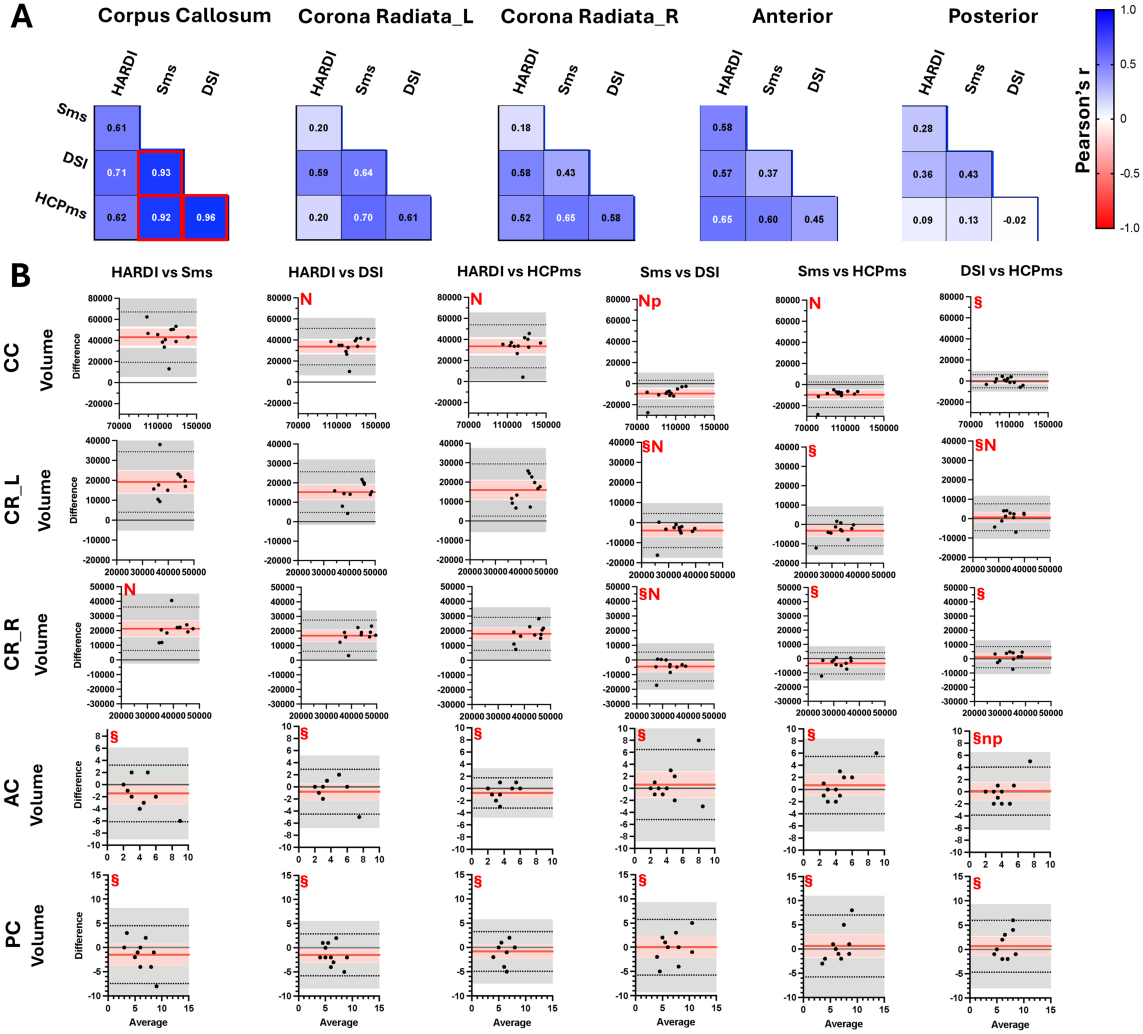
Correlation of tract-based volumes from four schemes in control group. Correlation of tract-based volumes from four schemes in control group plots show pair-wise comparison of tractography-based volume metrics from four schemes acquired in controls. **(A)** Pearson correlations of volumes of five white-matter tracks and **(B)** BA plots showing pair-wise comparisons. Red box, significant at Bonferroni-adjusted *p* < 0.05; L, left hemisphere; R, right hemisphere; HCP, HCPms; CC, corpus callosum; CR-L, corona radiata left hemisphere; CR-R, corona radiata right hemisphere; AC, anterior commissure; PC, posterior commissure; ^§^mean difference not significant at Tukey-adjusted *p* < 0.05; n, raw differences not normally distributed, but percent normally distributed; N, raw differences and percent not normally distributed; p, slope of the difference indicates proportional bias for raw scores but not for percent difference.

In controls, tract-based CC volumes from Sms correlated significantly with those from DSI and HCPms (Figure 9A), yet BA analysis (Figure 9B) showed that Sms consistently produced lower CC volume measurements relative to all other schemes. The opposite pattern was evident in Sms-derived volumes from the left and right CR: although not significantly correlated, they nonetheless agreed on average with both DSI and HCPms. Descriptive tract volume values are provided in Supplementary Table S6.

Likewise, in comparisons of DSI and HCPms, CC and both CR volumes were not significantly correlated but were in close agreement. However, HARDI volumes in CC and both CR ROIs were not significantly correlated and were consistently higher than all other schemes (Figure 9B). As described in Supplementary Figure S11, in comparisons of CC and CR tract-based volumes, with one exception (HARDI vs. HCP in CC) instances of non-normality were the result of a negative relationship between variance of differences and average volume.

In contrast to the variability across schemes and ROIs evident in CC and CR tract-based volumes, comparisons of tract-based volumes in the AC and PC were not significantly correlated but were consistent across all four schemes on average (AC *F*(2.115, 21.15) = 1.581, *p* = 0.2288, R2 = 0.1365; PC *F*(2.492, 24.92) = 1.389, *p* = 0.2695, *R^2^* = 0.1220; Figure 9B). Full repeated-measures ANOVA results are reported in Supplementary Table S2.

#### 3.3.2 Streamlines and streamline/volume

**Figure 10:**
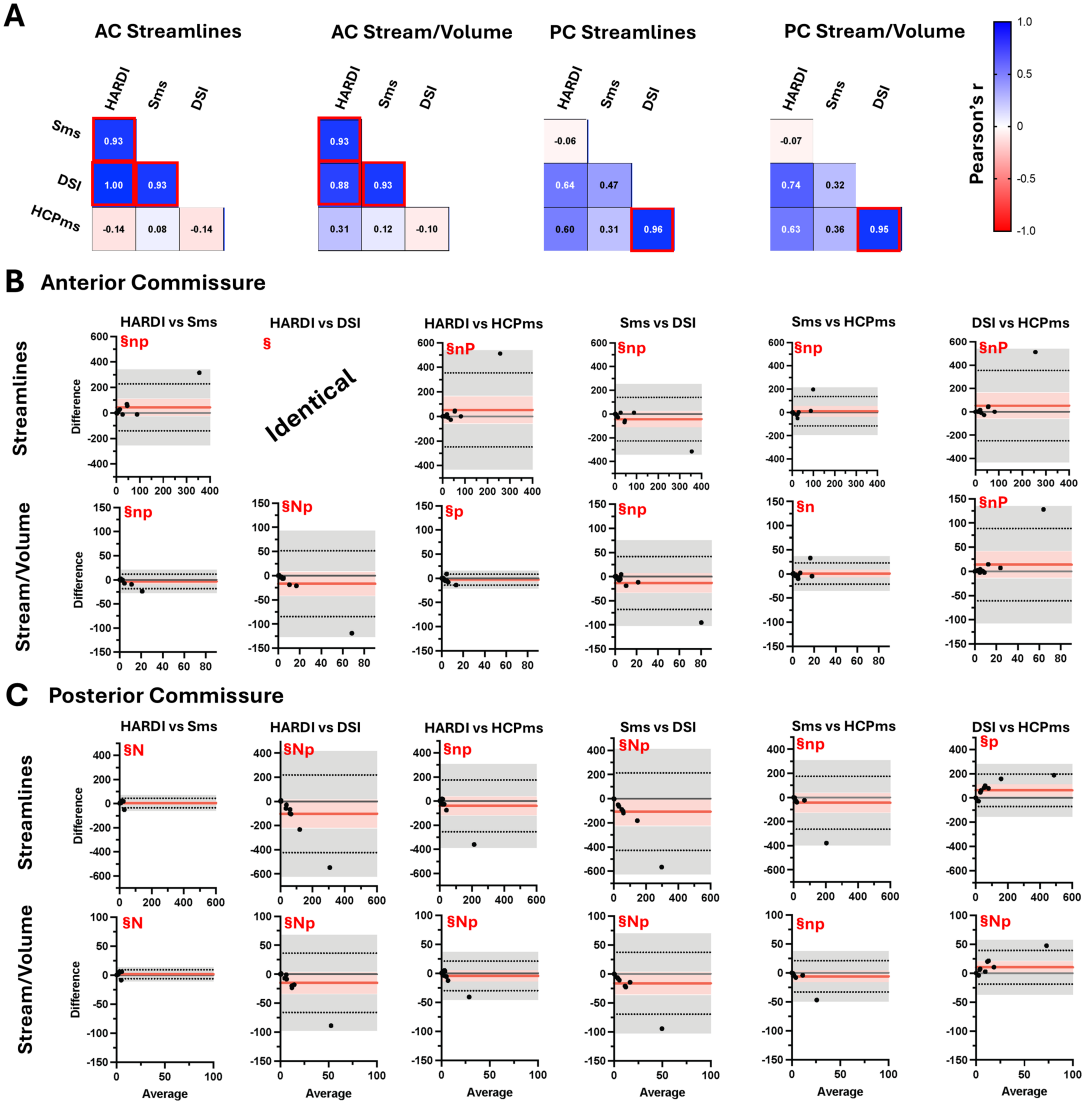
Correlation of tract-based streamline counts and streamline/volume from four schemes in control group. Bland–Altman plots comparing anterior and posterior commissure tractography metrics from four schemes. **(A)** Pearson correlations of tractography metrics from anterior commissure (AC) and posterior commissure (PC). Bland–Altman plots showing comparison of tractography metrics from four schemes for anterior commissure **(B)** and posterior commissure **(C)**. Black dots represent individual participant data (average value from the two methods on x-axis versus difference between values from the two methods on y-axis). Red lines show mean difference, with 95% confidence interval in pink. Dotted lines show limits of agreement with precision estimates (95% confidence interval) in gray. Equality value (0) is shown by a horizontal gray line. Subplots are scaled consistently across metric. Red box, correlation significant at Bonferroni-adjusted *p* < 0.05; ^§^mean difference not significant at Tukey-adjusted *p* < 0.05; n, raw differences not normally distributed, but percent normally distributed; N, raw differences and percent not normally distributed; p, slope of the difference indicates proportional bias for raw scores but not for percent difference.

Across the AC, streamline counts and streamline-to-volume ratios from HARDI, Sms, and DSI were all significantly correlated, whereas only DSI and HCPms showed significant correlations in PC metrics (Figure 10A). BA plots indicate that all four diffusion schemes yield consistent tract-based streamline metrics for both AC (streamline *F*(1.028, 10.28) = 1.516, *p* = 0.2468, *R*^2^ = 0.1316; streamline/volume *F*(1.040, 10.40) = 1.987, *p* = 0.1882, *R*^2^ = 0.1658) (Figure 10B) and PC (streamline *F*(1.105, 11.05) = 3.908, *p* = 0.0707, *R*^2^ = 0.281; streamline/volume *F*(1.094, 10.94) = 3.542, *p* = 0.0843, *R*^2^ = 0.2616) (Figure 10C). However, these outcomes must be interpreted cautiously, as robust statistical comparisons were limited by missing data and extreme outliers. At the participant-level, AC streamline detection failed in six participants, and PC streamline detection also failed in six participants. At the scheme-level, failures were observed in multiple protocols, with overlaps across schemes (i.e., the same participant could show zero streamlines in more than one acquisition): AC—DSI = 2, HARDI = 1, HCPms = 3, and Sms = 4 (Figure 10B); PC—DSI = 2, HARDI = 0, HCPms = 3, Sms = 6 (Figure 10C). In total, 8 out of 11 participants had at least one zero-streamline count, four with at least one zero result in both AC and PC. Because tract volumes are derived from the tractogram, zero-streamline cases also translate into zero-volume tract masks.

While most participants with a zero-streamline count also showed low streamline counts in other diffusion schemes, there were several exceptions. Most notably, in the AC the HCPms scheme failed to detect any streamlines in the participant who had the highest AC streamline counts for all three other schemes. Additionally, one participant had no AC streamlines from any of the schemes and only 4 PC streamlines identified by HARDI but had the second highest average PC streamline count for the other schemes. These frequent zero counts and select participant-specific inconsistencies between schemes appear to account for non-normality and proportional bias in the streamline metrics (Supplementary Figure S12). See Supplementary Table S4 for proportional bias results in AC/PC streamline metrics.

#### 3.3.3 CCD Tractography-based volume metrics

**Figure 11:**
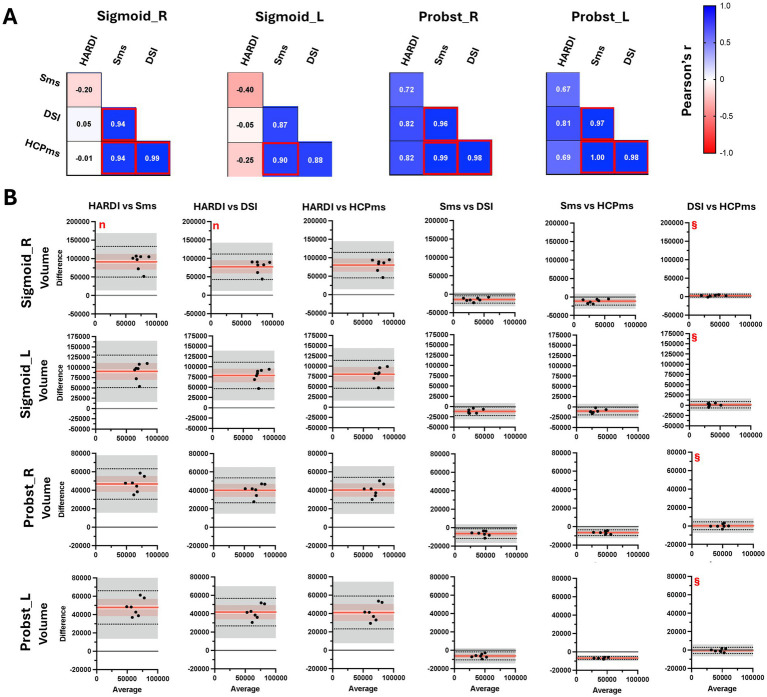
Correlation of tract-based volume of the Probst and sigmoid bundles from four schemes in CCD group. Plots show pair-wise comparison of sigmoid and Probst bundle volume (number of voxels) derived from four schemes. **(A)** Pearson correlations. **(B)** Bland–Altman plots. Black dots represent individual participant data (average value from the two methods on the x-axis versus difference between values from the two methods on the y-axis). Horizontal red lines show the mean difference, with 95% confidence interval in pink. Dotted horizontal lines show limits of agreement with precision estimates (95% confidence interval) in grey. The equality value (0) is shown by a horizontal black line. All subplots scaled consistently. Red box, correlation significant at Bonferroni-adjusted *p* < 0.05; ^§^mean difference not significant at Tukey-adjusted *p* < 0.05; n, raw differences not normally distributed, but percent normally distributed.

Tractography-based volume measurements derived via Sms, DSI, and HCPms, were significantly correlated for the right and left Probst and the right sigmoid bundle. However, only Sms and HCPms volumes were significantly correlated in the left sigmoid bundle (Figure 11A). Consistent with our control-group findings in CC and CR, the DSI and HCPms schemes yielded consistent volume estimates for both Probst and sigmoid bundles in each hemisphere. In contrast, Sms systematically produced lower volumes estimates relative to the other schemes, while HARDI produced higher estimates (Figure 11B). Full repeated-measures ANOVA results for tract-based volumes in CCD are provided in Supplementary Table S3.

## 4 Discussion

This study systematically compared four diffusion sampling schemes—HARDI, Sms, DSI, and HCPms—using identical hardware and processing pipelines to evaluate their impact on quantitative diffusion metrics and tractography outcomes in both typical and atypical brains. Water diffusion in white-matter is intrinsically non-Gaussian, with microstructural hindrance and restriction causing simpler tensor models to fail, especially in regions of complex fiber geometry. By sampling multiple b-values (DSI, HCPms, and to a lesser extent Sms), advanced diffusion sampling schemes capture both hindered diffusion (slowed by extracellular obstacles) and restricted diffusion (confined within cellular compartments), resulting in more comparable FA, MD, dispersion, and eCNR estimates and more consistent streamlines from tractography reconstructions when compared to single-shell HARDI.

### 4.1 Control subjects

#### 4.1.1 Diffusion properties

In healthy adults, we evaluated diffusion metrics in three white-matter ROIs: Corpus Callosum (CC), which has a well-established principal latero-lateral fiber orientation along the midline (Goldstein et al., 2022); Corona Radiata (CR), characterized by a predominant dorso-ventral fiber orientation (Kwon et al., 2014); and Centrum Semiovale (CSO), where cingulum, callosal, and CR fibers intersect (Yamada et al., 2007)—making it especially challenging for tractography.

Our findings demonstrate that diffusion metrics in the CC, CR, and CSO vary systematically with the choice of diffusion sampling scheme. While identifying a definitive “ground truth” protocol is beyond our scope (see section 5.5 Limitations), data acquired under different diffusion sampling schemes cannot be directly compared. These discrepancies arise from how the q-space is sampled—not from regional anatomy—underscoring a critical caveat for quantitative white-matter studies: even minor protocol variations on the same scanner can introduce significant bias. Although our findings warn against direct comparison of FA and MD values acquired with different diffusion schemes, the reported patterns of systematic bias between-schemes may be useful for identifying potential dataset anomalies or confounds to cross-scheme analysis.

#### 4.1.2 Crossing fibers

We examined each scheme’s ability to detect crossing fibers. In the highly anisotropic CC—often assumed to be a monofiber tract where the fibers are mainly oriented in the latero-lateral direction—we found a different number of 2-way crossing fibers (number of voxels with two different fiber orientations) but not with 3-way crossing fibers, which is likely due to the high CC anisotropy. Consistent with these observations, our voxel-wise BEDPOSTX analysis (Supplementary Figure S10) shows that scheme choice significantly modulates the proportion of multi-fiber voxels. The CC exhibits strong scheme effects for 2-way crossing fiber voxels but not for 3-way crossing fiber voxels at conservative thresholds, likely reflecting its high anisotropy and relatively homogeneous principal orientation. For the CSO and CR—regions rich in fanning and crossing fibers—inter-scheme differences were detected not only for 2-way fibers but also for 3-way. Our findings are in accordance with previous studies (Szczupak et al., 2020). In the CR and CSO, where multiple bundles intersect (Mollink et al., 2017), each diffusion scheme estimated a different prevalence of multi-fiber voxels, reflecting differences in angular resolution, maximum b-value, and SNR. Together, these results reinforce that diffusion metrics and tractography outcomes cannot be naively merged across protocols; inter-protocol differences in *q-space* sampling intrinsically alter crossing-fiber estimates.

#### 4.1.3 Tractography

To assess how diffusion sampling-dependent differences in crossing-fiber estimation affect connectivity tractography results, we applied identical seed and inclusion ROIs to reconstruct the CC and bilateral CR in each subject, then compared tract volumes. Our analyses revealed pronounced discrepancies in CC reconstructions across diffusion schemes, demonstrating that acquisition strategy can substantially alter tractography outcomes.

The CC is notoriously difficult to track: its fibers span the entire cortex (Goldstein et al., 2022) and intersect with numerous crossing-fiber voxels, often producing spurious streamlines. Therefore, the pronounced differences we observed in tractography outputs across protocols underscore how diffusion sampling schemes critically shape the fidelity of complex tractography.

Similarly, HARDI—despite using only a single b-value—produced larger CR volumes than other schemes. This may be attributed to the characteristics of the HARDI scheme, offering higher angular resolution which can better capture fiber orientations in regions of moderate complexity. As Dell’Acqua and Tournier (2019) showed, tractography accuracy depends less on the number of shells and more on angular resolution, maximum b-value, and image noise, all of which influence the shape of the orientation distribution function (ODF) and, consequently, the reconstructed pathways.

The higher angular resolution and appropriate b-values in the HARDI protocol enhance the ability to resolve fiber crossings, yielding more extensive tract reconstructions. Therefore, the larger bundle volumes observed with the HARDI scheme could be due to its superior capability to depict complex fiber configurations—evidence supported by previous studies (Dell’Acqua and Tournier, 2019; Tournier et al., 2013).

We also visually inspected the spatial overlap between tract reconstructions and noticed large discrepancies across protocols, even when tract volumes were comparable. This variation—independent of bundle identity and driven by diffusion sampling scheme—demonstrates that streamline trajectories can diverge markedly despite having similar overall volume. Such inconsistencies carry serious implications for connectome analysis, connectivity profiling, and studies of anatomical trajectories and anatomical alterations: protocol-dependent differences in reconstructed pathways may confound comparisons unless diffusion acquisition parameters are carefully controlled.

In addition, we also measured the volume of the AC and PC in the controls. These small compensatory commissures have become focal points in studies of congenital malformations (Tovar-Moll et al., 2014) and compensatory brain reorganization (Siffredi et al., 2021) offering sensitive markers of developmental plasticity. AC and PC exhibited varying streamlines across all diffusion sampling schemes. For example, one of the subjects showed zero-count streamlines for the anterior commissure in all schemes, suggesting a particularly challenging anatomy to trace. In contrast, three other subjects exhibited nonzero streamlines across all schemes except Sms, indicating a potential methodological limitation in that specific protocol. These findings underscore the combined influence of anatomical variability and acquisition-specific factors on the tractography of small commissures.

### 4.2 CCD participants

Most diffusion-MRI studies target neurotypical cohorts, leaving unaddressed the issue of how diffusion schemes perform in the presence of congenital malformations or brain injuries. By including CCD participants—whose Probst and sigmoid bundles reflect developmental rewiring—we evaluated each protocol’s generalizability and sensitivity to atypical anatomy. Protocols that exhibit consistency of tract metrics in both healthy and CCD brains demonstrate superior robustness and suitability for multicenter investigations spanning varied populations. This strategy not only validates the reliability of advanced diffusion methods but also enhances our understanding of how neurodevelopmental disruptions reshape white-matter connectivity. Analyses in CCD were not intended to test developmental effects. We restricted inferences to within-subject differences across schemes, refraining from between-participant or age-based conclusions. Taken together, these within-subject CCD results indicate that the observed between-scheme differences primarily reflect protocol choice in the context of atypical wiring, providing actionable guidance for protocol selection/harmonization in clinical and multicenter studies, rather than developmental effects.

We extended our tractography analysis to patients with CCD to probe each diffusion scheme’s performance in the presence of substantial anatomical rewiring. CCD is characterized by whole-brain reorganization (Wahl et al., 2009) and the emergence of aberrant long-distance pathways. The most well-studied examples of long-distance plasticity in these patients are the Probst and the Sigmoid bundles. The Probst bundle—comprised of callosal axons that failed to cross the midline (Ren et al., 2007)—and heterotopic sigmoid bundle—connecting the frontal cortex to contralateral parieto-occipital regions (Szczupak et al., 2020)—are hallmark examples of developmental plasticity in these individuals. Both bundles traverse the callosal remnant and are present only in cases of hypoplasia or partial dysgenesis, not in CC agenesis. Rather than judging one protocol’s absolute accuracy, our aim was to characterize how each diffusion sampling scheme influences the reconstruction of these unique fiber tracts.

Abnormal bundles are inherently challenging to reconstruct via tractography. These fibers fasciculate with pre-existing white matter tracts to find new pathways, usually resulting in portions of these bundles “kissing” fibers and other portions crossing these fibers. For example, the sigmoid bundle originates in the anterior pole, fasciculate (“kiss”) with the cingulum bundle while traveling posteriorly and crosses the midline after reaching the callosal remnant (crossing) (Tovar-Moll et al., 2007). Therefore, the tractography of abnormal bundles requires precise fiber orientation estimations and can be influenced by the diffusion sampling parameters. To test this hypothesis, we reconstructed the sigmoid and Probst bundles in each CCD participant and calculated their volumes under every diffusion scheme. Each sampling scheme produces bundles of varying size and anatomical extent, demonstrating that these protocols are not directly equivalent. This variability was consistent in both the sigmoid and Probst bundles, paralleling the differences seen in healthy controls. These results bring an important discussion on the mapping of abnormal bundles, especially in rare diseases. Due to the low incidence in the general population, these scientific reports commonly are done in case reports which utilize various clinical diffusion samples. The variety of diffusion MRI techniques associated with the high anatomical variability and the low sample size generates uncertainty on how different neurological disorders can affect the white matter connectivity.

### 4.3 Harmonization of dMRI

Although ComBat-like methods effectively reduce scanner- and site-specific biases in diffusion metrics, they assume comparable diffusion sampling schemes and primarily address additive or multiplicative effects (Fortin et al., 2017; Reynolds et al., 2023; Richter et al., 2022). Consequently, when protocols differ fundamentally, metric-level ComBat adjustments are unlikely to recover information not sampled by the acquisition. ComBat-GAM (to accommodate non-linear covariates, e.g., age) and Longitudinal ComBat (for repeated measures) extend the framework but retain the same comparability assumption (Beer et al., 2020; Pomponio et al., 2020).

At the signal level, rotation-invariant spherical harmonics (RISH) mapping and related approaches reduce cross-scanner differences by learning transforms in the spherical-harmonic domain (Mirzaalian et al., 2015, 2016, 2018). Dictionary-learning methods achieve similar goals without requiring paired data (St-Jean et al., 2020). Benchmark studies across scanners and across protocols—including multi-shell settings—show that these signal-level and deep-learning methods can compress inter-scanner variance, yet performance degrades as protocol differences increase, and many methods rely on training data with matched or partially matched protocols (Ning et al., 2020). A recent study evaluated how different machine learning–based approaches address cross-scanner and cross-protocol variability in diffusion MRI data (Tax et al., 2019).

While these algorithms effectively reduced cross-scanner variability, their performance relied on the availability of subjects with comparable acquisition protocols. Importantly, differences in angular and spatial resolution between protocols remain a significant challenge for harmonization. Addressing these intrinsic discrepancies will require harmonization methods designed specifically for different diffusion schemes—an important and still emerging area of research (De Luca et al., 2022; Zhong et al., 2020). In line with this, our analyses contrast schemes within-subject on the same scanner, and we therefore did not apply *post hoc* harmonization.

### 4.4 Implications and future directions

Our results emphasize that the diffusion sampling scheme is a fundamental source of variability in dMRI studies. While harmonization methods like ComBat adjust for scanner and site effects under the assumption of similar sampling, they may not fully reconcile differences arising from fundamentally distinct acquisition strategies. Accordingly, novel harmonization frameworks tailored to non-Gaussian diffusion models are needed. To further mitigate inter-scheme discrepancies, investigators should harmonize heterogeneous datasets by restricting analyses to common diffusion shells or applying correction techniques. Although we did not apply a formal linear-regression harmonization in this work, the tight correlations and low variance observed between Sms/HCPms, and other schemes suggest that a regression-based correction could effectively reduce systematic bias. Future studies should implement and validate such an approach to facilitate pooled analyses across diverse diffusion-acquisition protocols. By grounding protocol selection in both empirical evidence and theoretical understanding of non-Gaussian diffusion, researchers can improve reproducibility, validity, and generalizability of tractography and diffusion-metric studies across diverse populations.

Our systematic comparison of four diffusion-weighted MRI protocols—single-shell HARDI, Siemens multi-shell, DSI, and HCP multi-shell—confirms that sampling schemes have a significant effect on diffusion metrics and tractography outcomes in both healthy controls and patients with CCD. Crucially, by sampling multiple b-values, these advanced schemes capture non-Gaussian diffusion behavior—characteristic of hindered and restricted water motion in brain tissue—thereby improving the accuracy of both voxel-wise metrics and streamline reconstructions.

Based on these findings, we recommend that research groups adopt diffusion protocols that both optimize data quality and align with commonly used schemes. To enhance comparability with legacy or multi-site datasets, researchers can restrict analyses to common subsets—such as one phase encoding direction or a single diffusion shell—and apply advanced harmonization methods tailored for non-gaussian sampling differences. Future work could apply regression-based harmonization—and other methods—to reduce bias and enable pooled analyses across protocols. These strategies allow meaningful integration of heterogeneous data without significantly compromising quality-based harmonization.

We recognize, however, that multi-center pooling may introduce biases from hardware, software, and sequence variations that can rival those arising from diffusion sampling schemes alone. Accordingly, researchers must weigh the benefits of increased sample size against potential protocol-driven discrepancies and, when possible, standardize acquisition parameters and equipment across sites.

In conclusion, by choosing diffusion schemes grounded in empirical evidence and by applying harmonization strategies to heterogeneous datasets, the neuroimaging community can pursue truly global, multicentric studies of white-matter connectivity while potentially preserving reproducibility, validity, and methodological consistency.

### 4.5 Limitations

While we are evaluating how different schemes generate various diffusion metrics and examining the degree of agreement between schemes as reflected by these metrics, we are not directly assessing the reliability or internal consistency of each individual scheme, as we had no *a priori* reason to treat one of them as a reference scheme. A potential approach to address this limitation would be to implement a systematic test–retest design for each scheme. Also, because our tract-volume analyses were limited to within-subject comparisons across schemes, volumes were not adjusted by intracranial volume; intracranial volume normalization would be appropriate for between-subject or group-level volume comparisons and will be addressed in future work. A larger sample size would allow for a more systematic investigation of the factors contributing to zero-count streamline results observed in some subjects. This would help disentangle the relative impact of anatomical variability versus scheme-specific limitations, providing a more comprehensive understanding of the robustness and reliability of commissural tractography across individuals. A further limitation is that tractography-based reconstruction may occasionally fail for small bundles (e.g., AC/PC), yielding zero-volume tract masks in some subjects. Therefore, AC/PC tract volumes should be interpreted cautiously and primarily as descriptive measures. An additional limitation is that diffusion schemes were acquired in a fixed, non-randomized order. Although each run was preceded by Siemens automatic system adjustments (~2 min between runs), residual time-dependent effects over the course of a long session may still influence some measures (e.g., eCNR). Accordingly, we recommend randomized acquisition order and (split-session) test–retest designs in future work.

## 5 Data and code availability

The datasets generated and analyzed during the current study are not publicly available due to institutional and ethical restrictions. However, access to the data may be granted upon reasonable request to the corresponding author, subject to the following conditions:

1. A formal data sharing agreement must be established between the institutions involved.
2. Approval for data sharing must be granted by our institutional ethics committee.
3. A brief research proposal must be submitted outlining the intended use of the data.

Co-authorship is not required for data access, provided the proposed use aligns with the original ethical approvals and the data sharing terms. Custom analysis scripts used for diffusion metric extraction and tractography quantification are available upon request. Access to the code may also be subject to the same data sharing agreement, to ensure it is used within the appropriate research context.

## Data Availability

The datasets supporting the conclusions of this article are not publicly available due to institutional and ethical restrictions; access may be granted upon reasonable request to the corresponding author, subject to ethics approval and a formal data sharing agreement.

## Footnotes

1
http://www.cmrr.umn.edu/multiband

2
https://dsi-studio.labsolver.org

3
http://www.mrtrix.org

4
https://fsl.fmrib.ox.ac.uk

5
https://dipy.org

6
https://www.graphpad.com

## Glossary

AC: anterior commissure
AP: anterior to posterior
ANOVA: analysis of variance
AWS: Amazon Web Services
BA: Bland–Altman
BEDPOSTX: Bayesian Estimation of Diffusion Parameters Obtained using Sampling Techniques
BA: Bland–Altman
BIDS: Brain Imaging Data Structure
CA: callosal agenesis
CC: corpus callosum
CCD: corpus callosum dysgenesis
CNR: contrast-to-noise ratio
CR: corona radiata
CSO: centrum semiovale
DKI: diffusion kurtosis imaging
DSI: diffusion spectrum imaging
DTI: diffusion tensor imaging
eCNR: effective contrast-to-noise ratio
EDDY: eddy-current/motion correction
FA: fractional anisotropy
FODs: fiber orientation distributions
HARDI: high angular resolution diffusion imaging
HCPms: Human Connectome Project multi-shell
HP: callosal hypoplasia
ICV: intracranial volume
iFOD2: second-order Integration over Fiber Orientation Distributions
JHU Atlas: Johns Hopkins University white-matter atlas
MD: mean diffusivity
MSMT-CSD: multi-shell, multi-tissue constrained spherical deconvolution
ODF: orientation distribution function
PA: posterior to anterior
PC: posterior commissure
QC: quality control
RF: radio frequency
ROI: Region of interest
Sms: Siemens multi-shell
TOPUP: susceptibility distortion correction
