# Supplementary material for: Diffusion MRI sampling schemes bias diffusion metrics and tractography
Source: Front Neuroimaging. 2026 Feb 25;5:1670604. doi: 10.3389/fnimg.2026.1670604 (PMC12975589; doi:10.3389/fnimg.2026.1670604)
Supplement: Supplementary file 1 [file Data_Sheet_1.pdf]

Supplementary Material

Figure S1. Comparison of corpus callosum tract reconstructions across four diffusion schemes. *Tractography was performed in DSI Studio by placing bilateral ROIs in the corpus callosum - left (red) and right (blue) (A) - and visualizing the resulting streamlines in the coronal, axial, and sagittal views. Panels B–E show reconstructions obtained with four different diffusion schemes: (B) DSI (diffusion spectrum imaging), (C) HARDI (high-angular resolution diffusion imaging), (D) HCPms (Human Connectome Project multi-shell), and (E) Sms (Siemens multi-shell).*

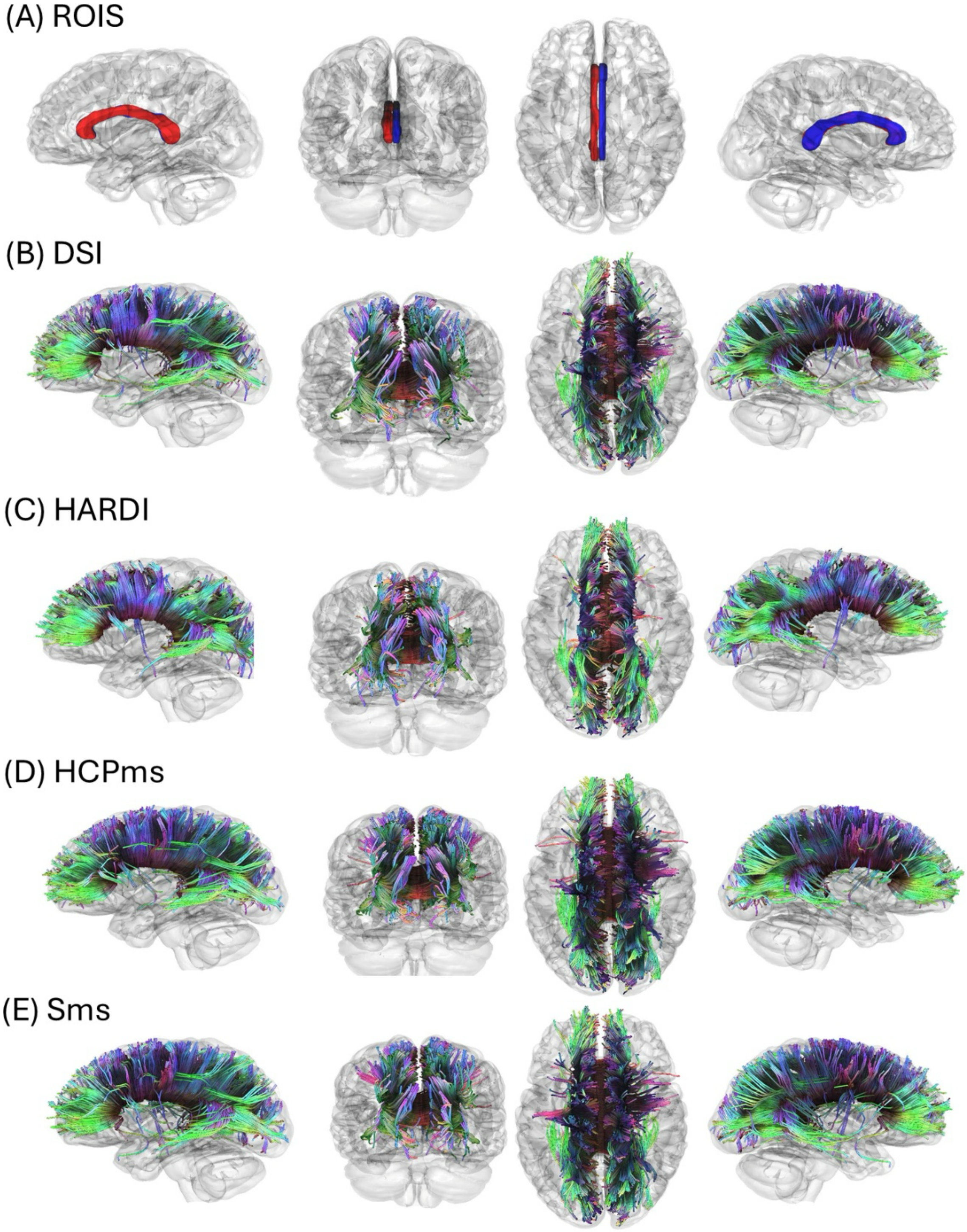

Figure S2. Comparison of corticospinal tract reconstructions across four diffusion schemes. *Tractography was performed in DSI Studio by placing inclusion ROIs in the corona radiata - left (red) and right (blue) - and in the lower brainstem at the level of the cerebral peduncles (dark blue) (A). The resulting streamlines are shown in the coronal, axial, and sagittal views. Panels B–E display reconstructions obtained with four different diffusion schemes: (B) DSI (diffusion spectrum imaging), (C) HARDI (high-angular resolution diffusion imaging), (D) HCPms (Human Connectome Project multi-shell), and (E) Sms (Siemens multi-shell).*

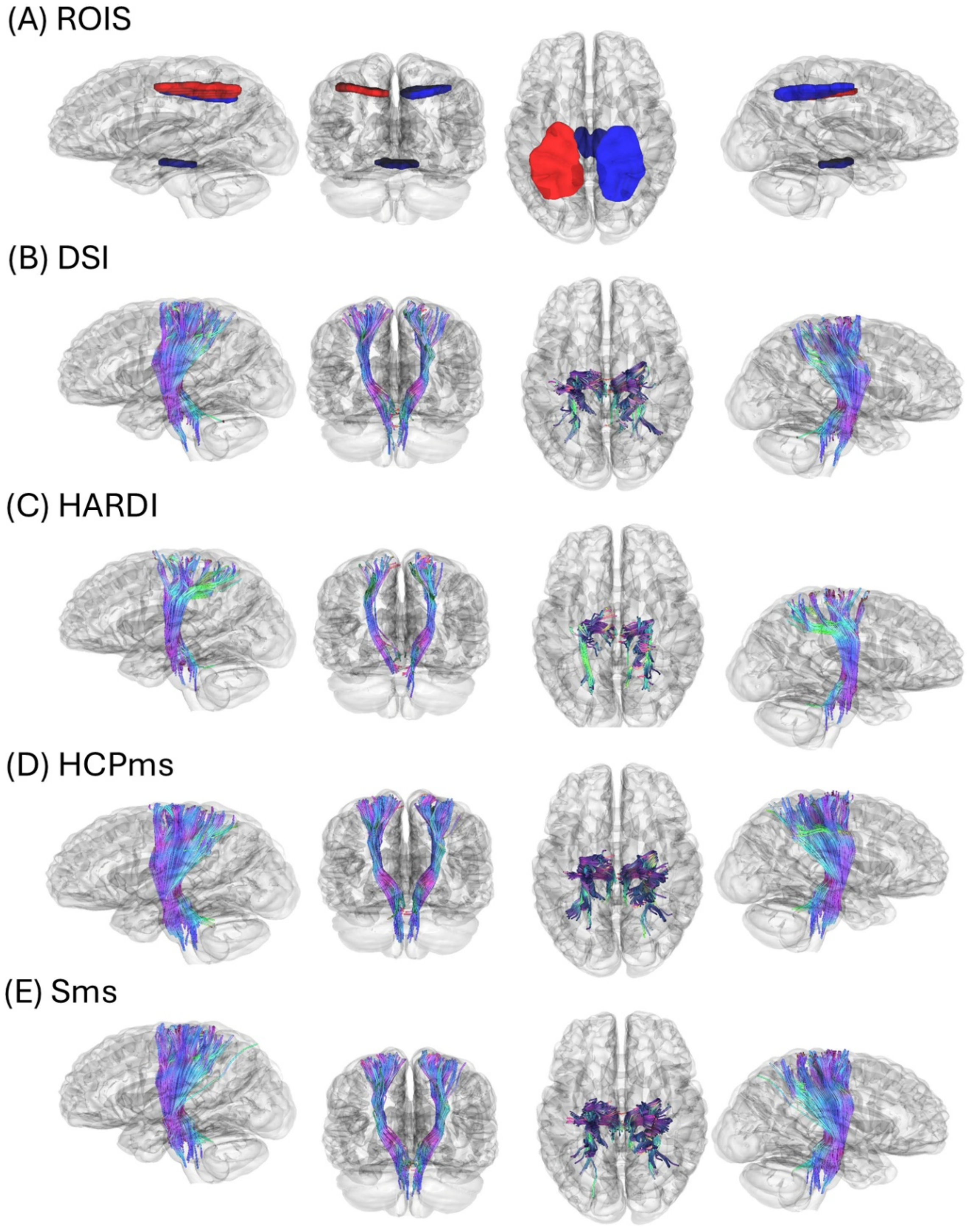

Figure S3. Comparison of Probst tract reconstructions across four diffusion schemes. *Tractography was performed in DSI Studio by placing two large inclusion ROIs in the coronal plane - left (pink) and right (blue) - one at the level of the bilateral cingulate bundles and another around the splenium to encompass the medial longitudinal fibers - and combining them ipsilaterally to isolate the Probst bundles (A). The resulting streamlines are shown in the coronal, axial, and sagittal views. Panels B–E display reconstructions obtained with four different diffusion schemes: (B) DSI (diffusion spectrum imaging), (C) HARDI (high-angular resolution diffusion imaging), (D) HCPms (Human Connectome Project multi-shell), and (E) Sms (Siemens multi-shell).*

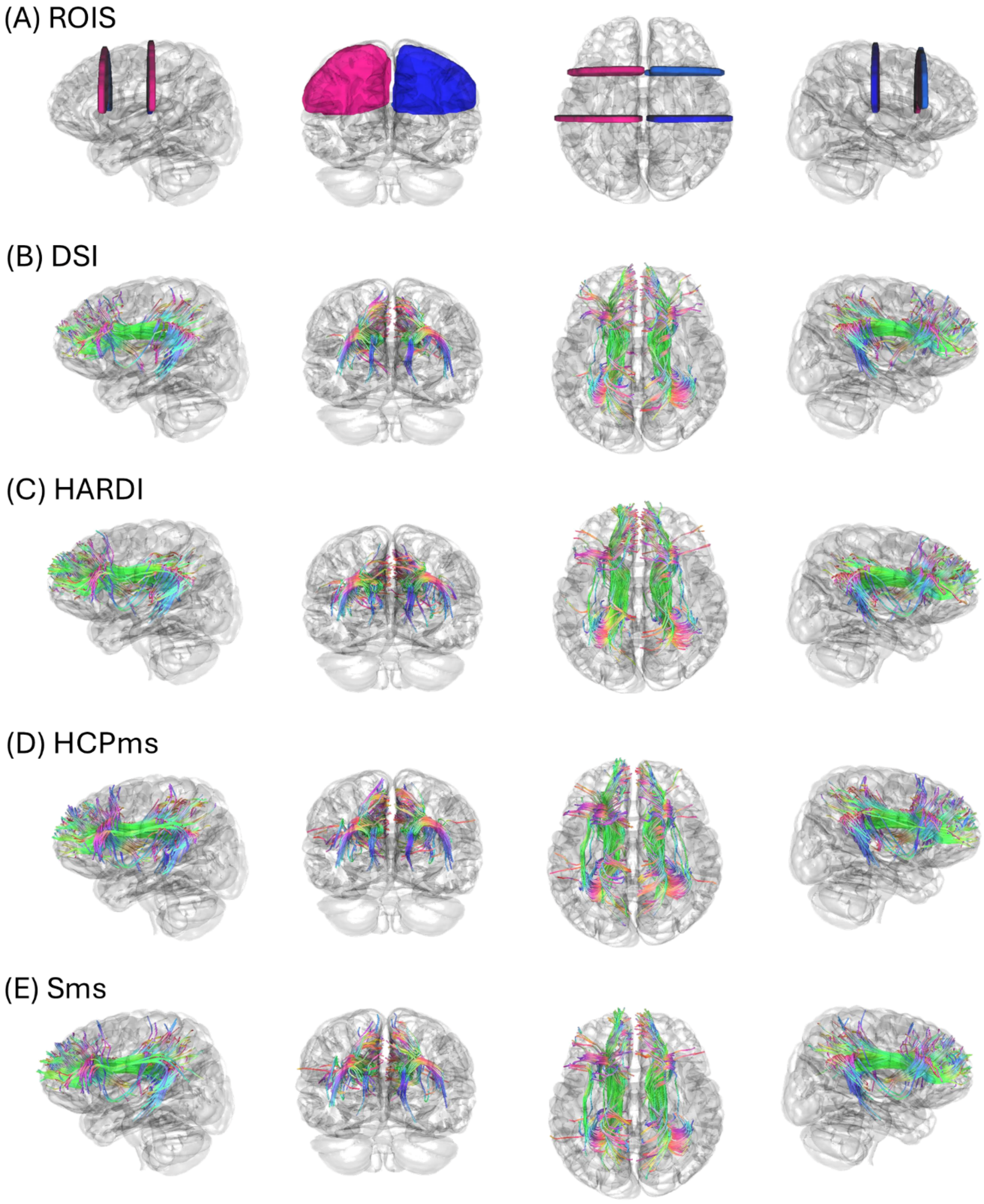

Figure S4. Comparison of sigmoid bundle reconstructions across four diffusion schemes. *Tractography was performed in DSI Studio by placing two large inclusion ROIs - left (pink) and right (blue) - in the coronal plane - one at the level of the bilateral cingulate bundles and another around the splenium to encompass the medial longitudinal fibers - and combining them contralaterally to isolate the sigmoid bundles (A). The resulting streamlines are shown in the coronal, axial, and sagittal views. Panels B–E display reconstructions obtained with four different diffusion schemes: (B) DSI (diffusion spectrum imaging), (C) HARDI (high-angular resolution diffusion imaging), (D) HCPms (Human Connectome Project multi-shell), and (E) SMS (Siemens multi-shell).*

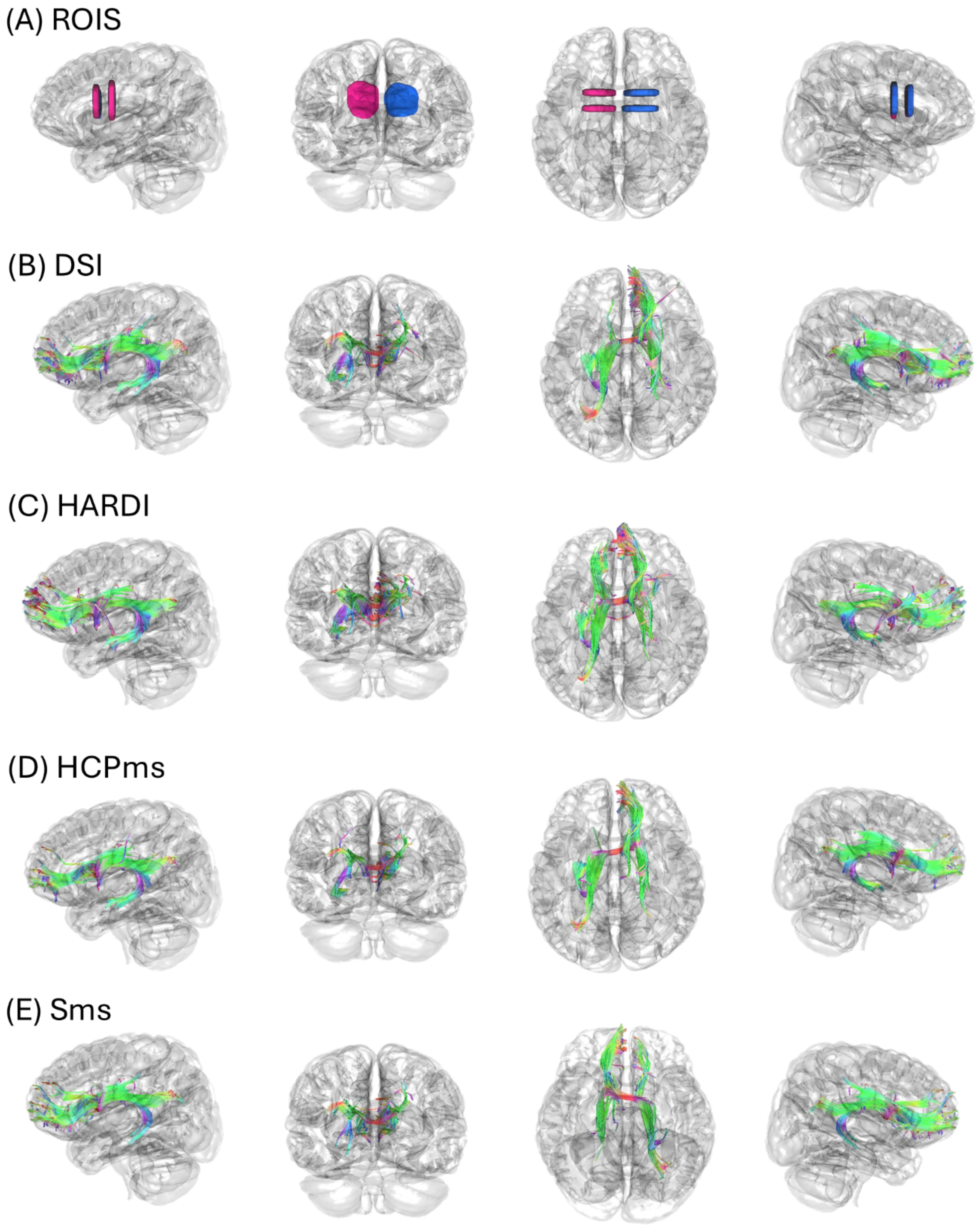

Figure S5. Post-hoc BA Plots of Percent Difference in Absolute Motion. *Raw score contrasts which were not normally distributed and/or indicated proportional bias were reexamined following conversion to percent difference. Percent difference between Sms vs. DSI (A) and HARDI vs. HCPms (B) was normally distributed without indication of proportional bias, as shown on standard BA plots of percent difference. In contrast, although normally distributed, proportional bias was evident in percent difference contrasts between Sms vs. HCPms 95%CI [-63.67, -15.87]; DSI vs. HCPms 95%CI [-56.27, -18.24]) and BA regression plots indicate that with increased absolute motion, differences from HCPms become greater for both Sms (C) and DSI (D).*

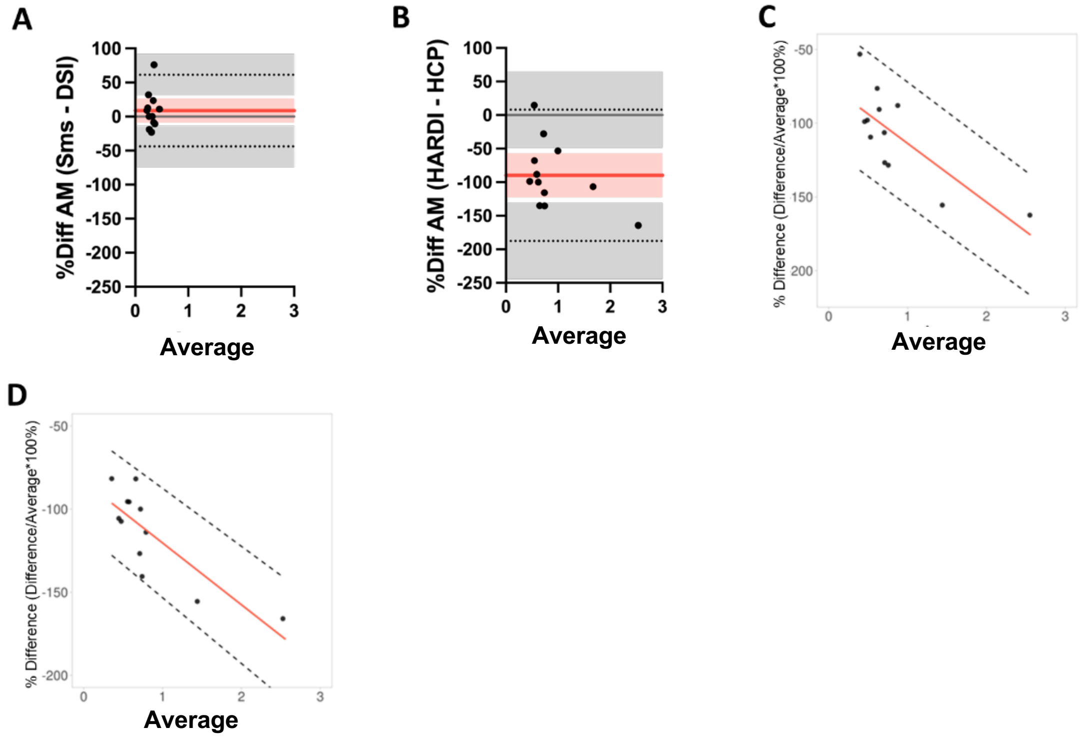

Figure S6. Post-hoc BA Plots of Relative Motion. *Raw score contrasts with HCPms indicated proportional bias (HARDI vs. HCPms, [-2.46, -1.31]; Sms vs. HCPms, [-2.08, -0.78]; DSI vs. HCPms, [-2.37, -0.82]), but conversion to percent difference either introduced non-normality (HARDI vs. HCPms) or did not eliminate proportional bias Sms vs. HCPms, [-303.12, -3.9], and DSI vs. HCPms, [-364.24, -18.16]. Thus, proportional bias was examined with BA regression plots of raw differences, indicating that HCPms outcomes become more similar to HARDI (A), Sms (B), and DSI (C) with increased relative motion.*

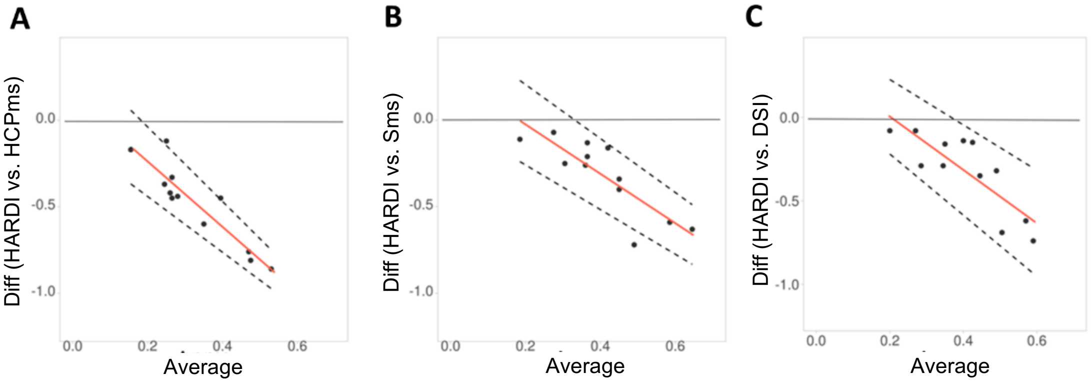

Figure S7. Post-hoc BA Plots of MD in CSO. *Non-normality and proportional bias were evident for all comparisons with HARDI (Sms [0.84, 1.57]; DSI [0.73, 1.23]; HCPms [0.92, 1.49]). Conversion to percent difference eliminated non-normality for these comparisons, but proportional bias remained (Sms [27995.1, 152504.07]; DSI [30942.3, 114529.72]); HCPms [46632.17, 144480.17]). BA regression plots A-C below indicate percent difference from HARDI increases as average MD increases (A, Sms; B, DSI, and C, HCPms). Comparison of Sms and DSI revealed non-normality for original scores and percent difference, and regression plot of original scores indicates that increased mean MD for the CSO is associated with greater variability and smaller mean difference between MD values from these schemes. (D). Proportional bias was also evident for raw score comparison of DSI and HCPms ([0.06, 0.46]) but was eliminated by conversion to percent difference (E).*

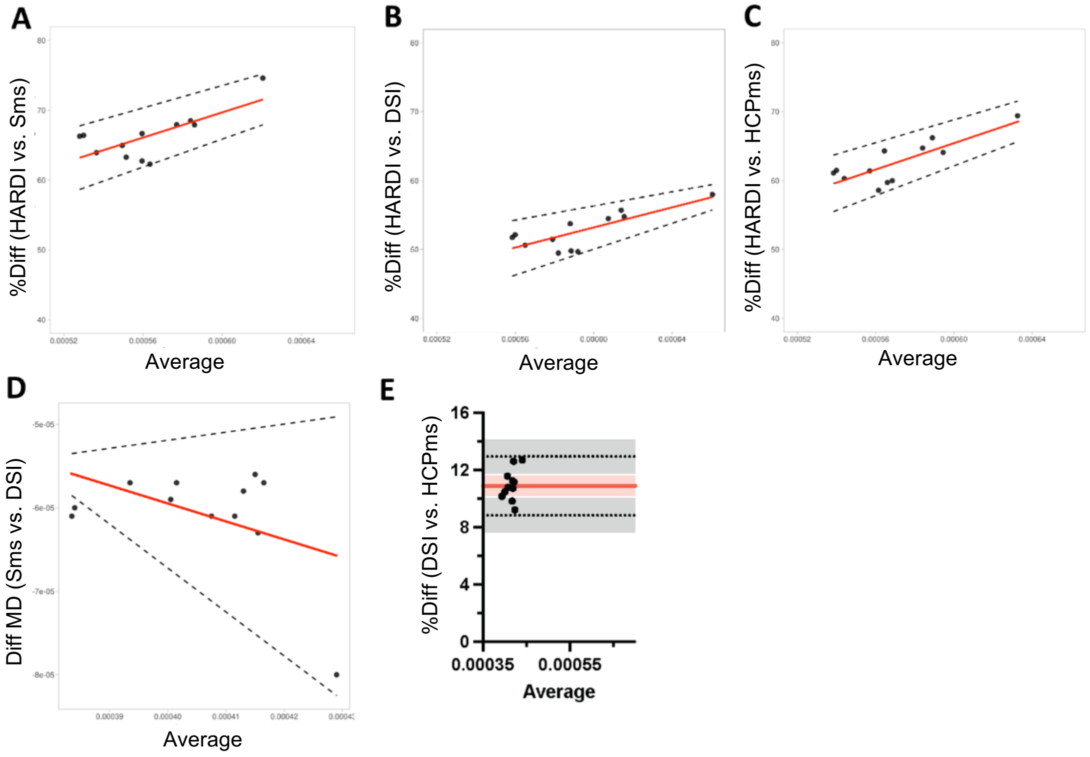

Figure S8. Post-hoc BA Regression Plots of eCNR in each region of interest. *For eCNR metrics in CSO, proportional bias was evident in the HARDI vs. DSI comparison with raw scores [1.34, 1.7] and percent difference [1.79, 5.8], due to positive association between average and difference of raw eCNR (A). Likewise, comparison of HARDI and HCPms revealed non-normality and proportional bias for both raw scores [0.76, 2.83] and percent difference [1.78, 19.7], with non-normality also reflecting positive association between the average and difference of raw eCNR and proportional bias reflecting positive association between the average and variability of differences (B). In all regions of interest, comparison of eCNR metrics from Sms vs. HCPms was not normally distributed for raw scores or percent difference. In CC (C) and CR (D), non-normality reflected a negative association between average eCNR and variability of differences (i.e., less variability as average increased). It was the opposite in CSO (E), which had a positive association between average eCNR and variability of differences (i.e., greater variability as average increased) in addition to evidence of positive association between average eCNR and average of differences. Comparison of raw eCNR from DSI vs. HCPms revealed non-normality and proportional bias for CR [-2.09, -1.04] and for CSO [-2.67, -0.43]. Following conversion to percentage, both non-normality and bias remained for CR [-36.98, -12.15], but only non-normality remained for CSO. Regression reveals a negative association between the average and difference of raw eCNR values from DSI and HCPms in both CR (F) and CSO (G), in addition to decreased variability of differences in CSO (G).*

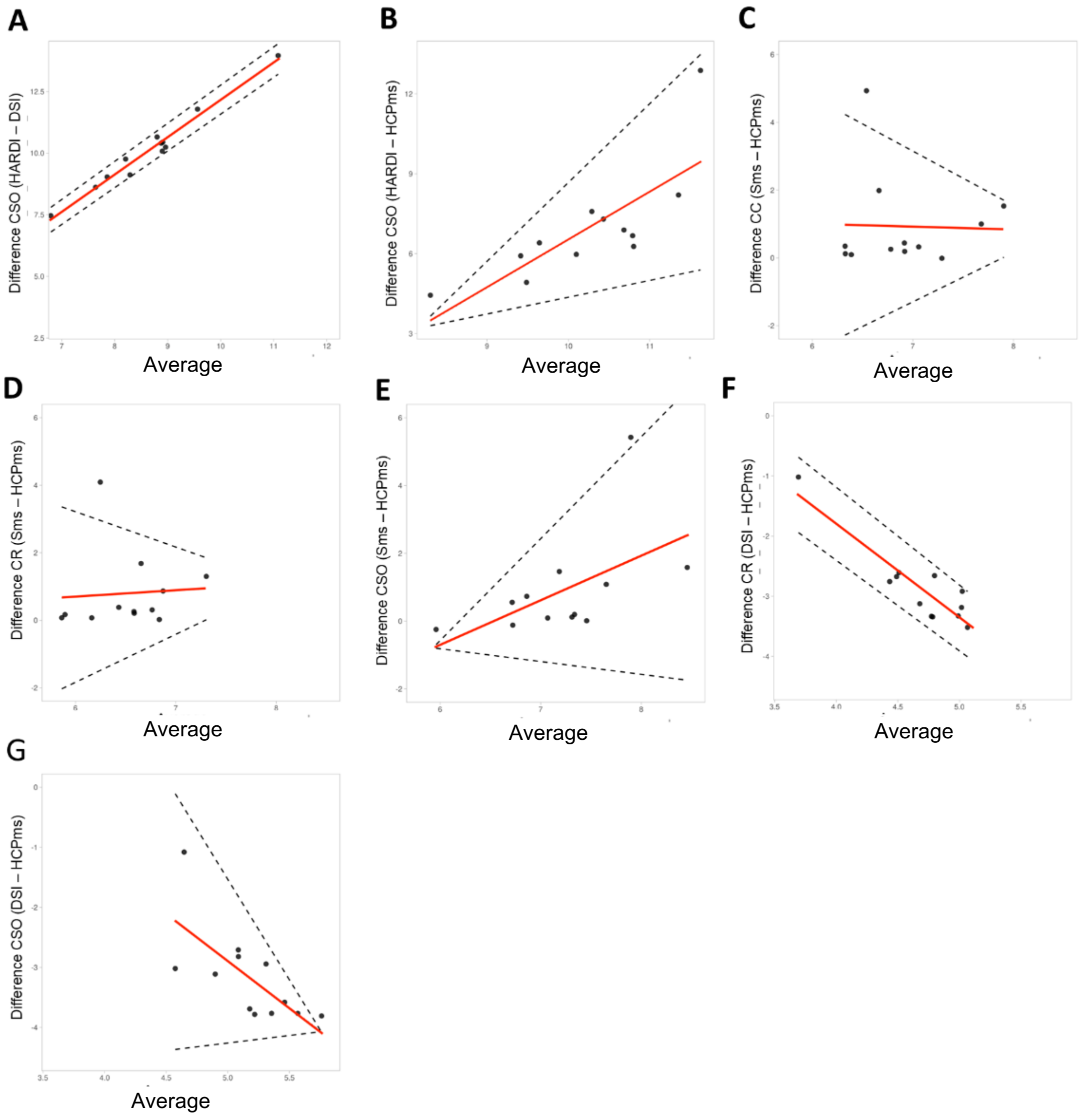

Figure S9. Post-hoc BA Plots of Dispersion in each region of interest. *Comparison of HARDI vs. Sms dispersion in CSO indicated proportional bias [-1.06, -0.48] which was eliminated following conversion to percent difference (A). Comparison of HARDI vs. DSI dispersion values in CC was not normally distributed for raw or percent difference due to positive association between average dispersion and variability of differences. (B). Comparison of HARDI vs. HCPms dispersion values in CC demonstrated non-normality and proportional bias [-2.84, -0.65] for raw and percent [-11111.65, -1864.8] due to greater variability and smaller mean raw difference with increased average dispersion values (C). For comparison of HARDI vs. HCPms in CSO (D) and Sms vs. DSI in CC (E), non-normality of both raw and percent differences was due to positive association between average dispersion and variability of differences. For comparison of Sms vs. DSI in CSO proportional bias was only evident with raw scores [0.06, 1.41], but non-normality for both raw and percent difference revealed a positive association between average dispersion and variability of differences, as well as a subtle negative association between average score and mean difference (F). Comparisons of DSI vs. HCPms in CC and CR were characterized by non-normality for raw scores and by proportional bias for both raw scores (CC [-1.11, -0.43] and CR [0-.59, -0.02]) and percent difference (CC [-3702.09, -758.59] and CR [-6477.85, -1198.63]), with regression indicating increased average dispersion was associated with greater variability and lower average of differences (G, H). In CSO, comparison of DSI and HCPms dispersion values was not normally distributed for raw or percent difference, due to positive association between average dispersion and variability of differences (I).*

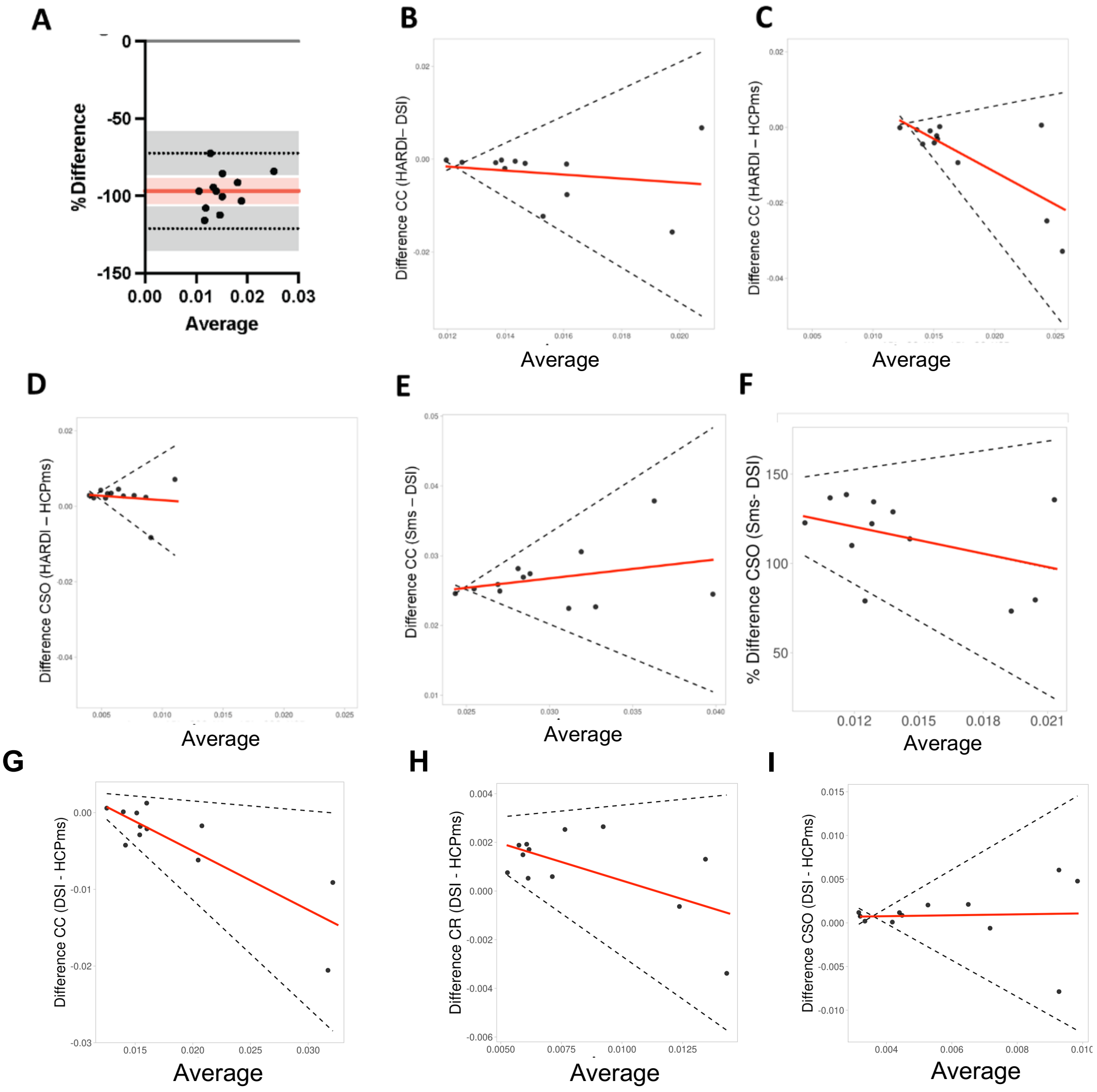

Figure S10. Multi-fiber voxel prevalence across diffusion schemes. *Percentage of voxels per ROI classified as 2-way fiber ($f_2 > 0.05$; $f_2 > 0.10$) and 3-way fiber ($f_3 > 0.05$; $f_3 > 0.1$) from BEDPOSTX mean of the probabilistic distribution of the anisotropic volume fraction maps. Rows show ROIs: corpus callosum (CC), corona radiata (CR), centrum semiovale (CSO); columns show thresholds. Boxplots summarize participant distributions for HARDI, Sms, DSI, and HCPms; percentages are relative to ROI size. Brackets indicate pairwise differences across schemes.*

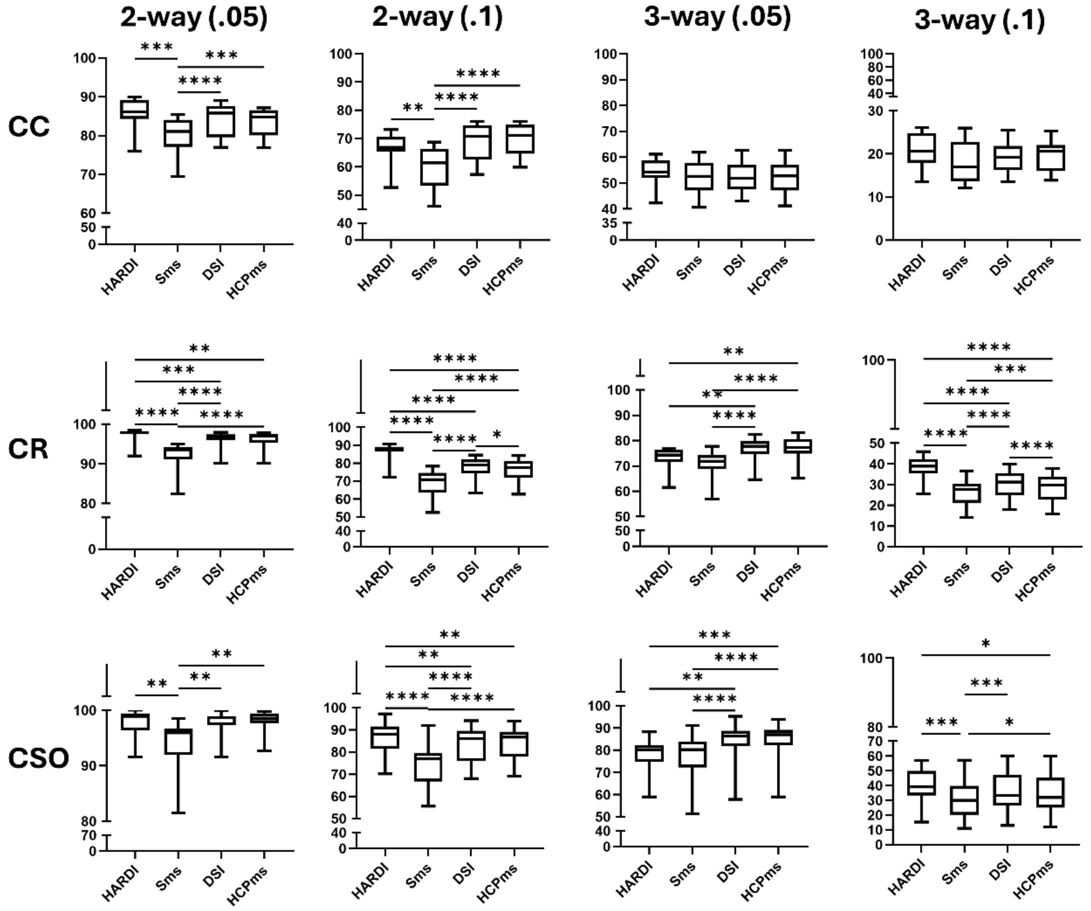

Figure S11. Post-hoc BA Plots of tract-based volumes in control participants. *Both raw and percent difference were non-normally distributed for comparison of CC volumes from HARDI vs DSI and HARDI vs HCPms, and for CR-R volumes from HARDI vs Sms. Regression plots of raw-score differences for HARDI vs. DSI in CC (A) and for HARDI vs. Sms in CR-R (B) reveal decreased variability with greater average volume, with the opposite pattern for HARDI vs. HCPms in CC (increased variability of differences with higher average volume, C). Comparison of Sms and DSI tract-based CC volumes demonstrated non-normality and proportional bias [0.1, 0.62], with non-normality remaining after conversion to percent difference and BA regression plots revealing a negative relationship between variance of differences and average CC volume (D). Both raw and percent difference of tract-based volume were non-normally distributed for comparison of Sms vs. DSI in CR-L (D) and CR-R (F), for Sms vs HCPms in CC (G), and for DSI vs. HCPms in CR-L (H), with BA regression plots revealing a negative relationship between variance of differences and average volume for each of these comparisons. Comparison of AC volumes from DSI vs. HCPms was not normally distributed and indicated proportion bias [0.02, 1.64], both of which were corrected by conversion to percent difference (I).*

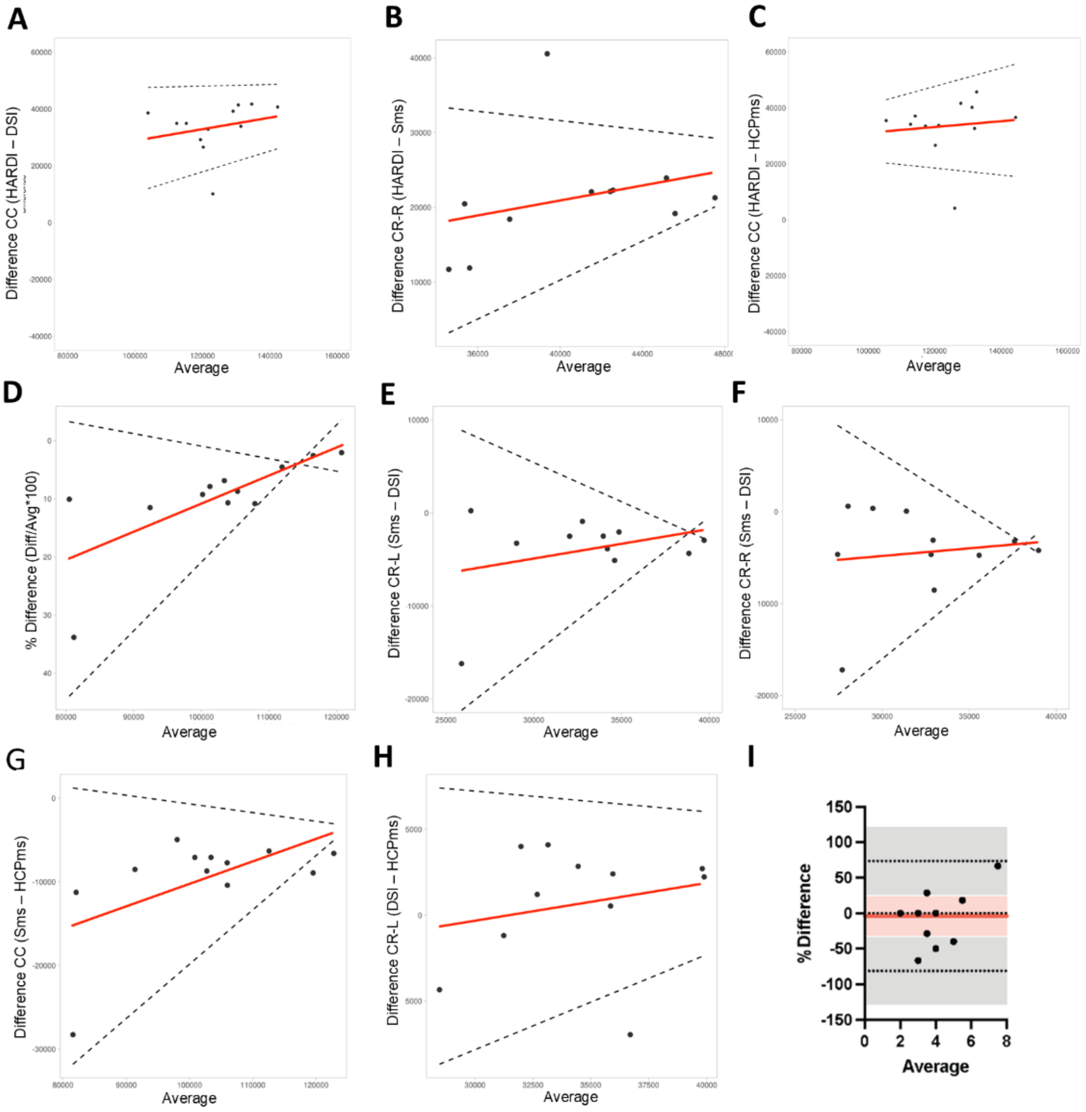

Figure S12. Post-hoc BA plots of AC and PC tract-based streamlines and streamlines/volumes. *As noted in the text, eight participants out of 11 had one or more zero streamline results on AC (n-2), PC (n=2) or both (n=4) and many comparisons also included an elevated outlier. HARDI vs. DSI identified an identical number of AC streamlines. Among the remaining raw score comparisons of AC tract-based streamlines and streamlines/volume, only HARDI vs. HCPms streamlines/volume were normally distributed and only Sms vs. HCPms streamlines/volume did not exhibit proportional bias (Table S1). Although conversion to percent difference corrected most of these instances of non-normality and proportional bias, for AC streamline/volume proportional bias remained for the comparison of DSI vs. HCPms [.1, 1.42] (B), and non-normal distribution appeared for HARDI vs. HCPms [.1, 1.42] (C), and non-normality remained for HARDI vs. DSI (D). Likewise for raw PC tract-based streamlines and streamlines/volume, only DSI vs. HCPms streamlines were normally distributed and only HARDI vs. Sms streamlines and streamlines/volume did not exhibit proportional bias (Table S1). Conversion to percent difference corrected all instances of proportional bias but introduced bias into comparisons of HARDI vs. Sms PC streamlines and streamlines/volume. Conversion also corrected normality in comparison of HARDI vs. HCPms streamlines, as well as Sms vs. HCPms streamlines and streamlines/volume. Follow-up BA regression plots indicate residual non-normality and proportional bias effects. The regression plots below (A-M) indicate non-normality and proportional bias in comparisons of tract-based AC and PC metrics was strongly influenced by a limited number of participants who had substantial variation across regions and metrics (e.g., a participant with streamline counts of 582 and 393 for DSI and HCPms respectively, and <34 for HARDI and Sms).*

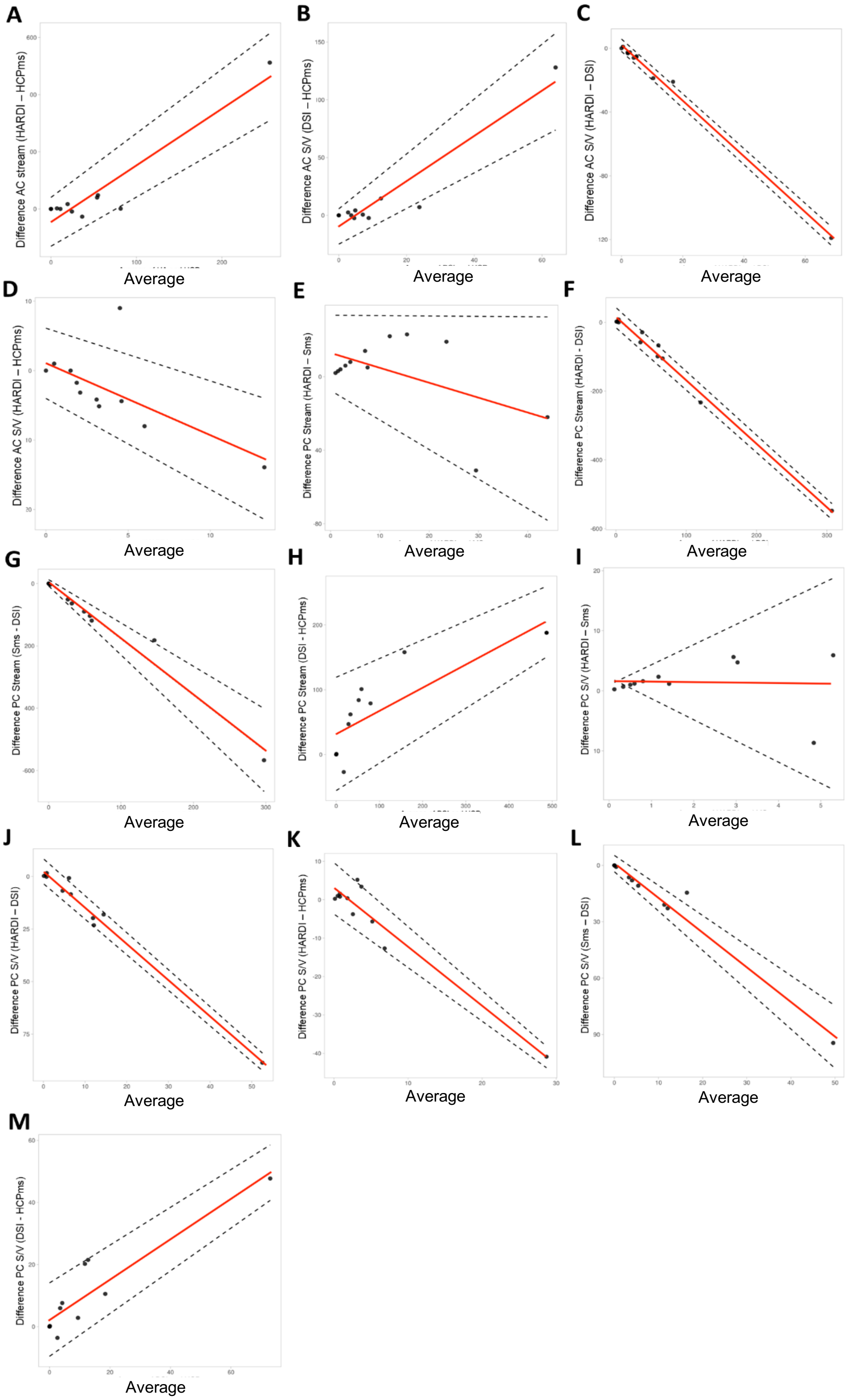
A
Difference AC stream (HARDI – HCPms)
Average
B
Difference AC S/V (DSI – HCPms)
Average
C
Difference AC S/V (HARDI – DSI)
Average
D
Difference AC S/V (HARDI – HCPms)
Average
E
Difference PC Stream (HARDI – Sms)
Average
F
Difference PC Stream (HARDI - DSI)
Average
G
Difference PC Stream (Sms - DSI)
Average
H
Difference PC Stream (DSI - HCPms)
Average
I
Difference PC S/V (HARDI – Sms)
Average
J
Difference PC S/V (HARDI – DSI)
Average
K
Difference PC S/V (HARDI – HCPms)
Average
L
Difference PC S/V (Sms – DSI)
Average
M
Difference PC S/V (DSI - HCPms)
Average

Table S1. Repeated measures one-way ANOVA comparison of diffusion metrics from 4 schemes in controls. *NOTE:* FA = fractional anisotropy; MD = mean diffusivity; eCNR = effective Contrast to Noise Ratio; % voxels = percent of voxels whose anisotropic volume fraction exceeds fixed threshold; CC = corpus callosum; CR = corona radiata; CSO = centrum semiovale.

**Measure**	**ROI**	**F (DFn, DFd)**	**P value**	**R^2**
Absolute motion	whole	F (1.070, 11.77) = 10.03	P=0.0076	0.4769
Relative motion	whole	F (1.319, 14.51) = 33.28	P<0.0001	0.7516
FA	CC	F (1.234, 13.58) = 138.5	P<0.0001	0.9264
FA	CR	F (1.231, 13.55) = 186.2	P<0.0001	0.9442
FA	CSO	F (1.246, 13.71) = 188.9	P<0.0001	0.945
MD	CC	F (1.014, 11.16) = 685.3	P<0.0001	0.9842
MD	CR	F (1.033, 11.36) = 1394	P<0.0001	0.9922
MD	CSO	F (1.042, 11.46) = 1314	P<0.0001	0.9917
eCNR	CC	F (1.539, 16.93) = 554.8	P<0.0001	0.9806
eCNR	CR	F (2.007, 22.08) = 389.7	P<0.0001	0.9725
eCNR	CSO	F (1.566, 17.23) = 196.2	P<0.0001	0.9469
dispersion	CC	F (1.370, 15.07) = 90.05	P<0.0001	0.8911
dispersion	CR	F (2.084, 22.92) = 163.2	P<0.0001	0.9369
dispersion	CSO	F (1.941, 21.36) = 92.22	P<0.0001	0.8934
% voxels 2-way (.05)	CC	F (1.423, 15.65) = 16.33	P=0.0004	0.5976
% voxels 2-way (.05)	CR	F (1.395, 15.35) = 77.87	P<0.0001	0.8762
% voxels 2-way (.05)	CSO	F (1.207, 13.28) = 19.69	P=0.0004	0.6416
% voxels 2-way (.1)	CC	F (1.443, 15.87) = 26.15	P<0.0001	0.7039
% voxels 2-way (.1)	CR	F (1.562, 17.18) = 180.9	P<0.0001	0.9427
% voxels 2-way (.1)	CSO	F (1.428, 15.71) = 70.27	P<0.0001	0.8647
% voxels 3-way (.05)	CC	F (1.213, 13.35) = 0.980	P=0.3579	0.0818
% voxels 3-way (.05)	CR	F (1.252, 13.77) = 51.38	P<0.0001	0.8237
% voxels 3-way (.05)	CSO	F (1.786, 19.64) = 28.44	P<0.0001	0.7211
% voxels 3-way (.1)	CC	F (1.691, 18.60) = 2.258	P=0.1383	0.1703
% voxels 3-way (.1)	CR	F (1.145, 12.59) = 97.23	P<0.0001	0.8984
% voxels 3-way (.1)	CSO	F (1.615, 17.77) = 15.86	P=0.0002	0.5904

Table S2. Repeated measures one-way ANOVA comparison of tract-based metrics from 4 schemes in controls. *NOTE:* CC = corpus callosum; CR-L = corona radiata left hemisphere; CR-R = corona radiata right hemisphere; AC = anterior commissure; PC = posterior commissure

**Measure**	**ROI**	**F (DFn, DFd)**	**P value**	**R^2**
tract-based volume	CC	F (1.562, 17.18) = 121.2	P<0.0001	0.9168
tract-based volume	CR-R	F (1.908, 19.08) = 68.95	P<0.0001	0.8733
tract-based volume	CR-L	F (1.729, 17.29) = 53.03	P<0.0001	0.8413
tract-based volume	AC	F (2.115, 21.15) = 1.581	P=0.2288	0.1365
Streamline count	AC	F (1.028, 10.28) = 1.516	P=0.2468	0.1316
streamlines/volume	AC	F (1.040, 10.40) = 1.987	P=0.1882	0.1658
tract-based volume	PC	F (2.492, 24.92) = 1.389	P=0.2695	0.1220
streamline count	PC	F (1.105, 11.05) = 3.908	P=0.0707	0.2810
streamlines/volume	PC	F (1.094, 10.94) = 3.542	P=0.0843	0.2616

Table S3. Repeated measures one-way ANOVA comparison of tract-based volumes from 4 schemes in CCD. *NOTE: ROI = region of interest; PB= Probst Bundle; R = right; L= left; Sig = Sigmoid bundle*

Measure	ROI	F (DFn, DFd)	P value	R^2
tract-based volume	PB-R	F (1.148, 6.890) = 210.9	P<0.0001	0.9723
tract-based volume	PB-L	F (1.040, 6.242) = 171.0	P<0.0001	0.9661
tract-based volume	Sig-R	F (1.089, 6.533) = 130.3	P<0.0001	0.9560
tract-based volume	Sig-L	F (1.112, 6.674) = 140.8	P<0.0001	0.9591

Table S4. Proportional bias in between-scheme comparisons of Anterior and Posterior Commissure tract-based streamline metrics. *NOTE: AC = anterior commissure; SL = streamlines; SL/V = streamlines by volume; PC = posterior commissure*

	**HARDI x Sms**	**HARDI x DSI**	**HARDI x HCPms**	**Sms x DSI**	**Sms x HCPms**	**DSI x HCPms**
**AC SL**	[0.62, 1.09]	NA	[1.46, 2.5]	[-1.09, -0.62]	[0.32, 2.34]	[1.46, 2.5]
**AC SL/V**	[-1.37, -0.97]	[-1.84, -1.65]	[-1.93, -0.14]	[-1.36, -0.97]	---	[1.48, 2.44]
**PC SL**	---	[-1.97, -1.7]	[-1.97, -1.57]	[-2.04, -1.57]	[-2.14, -1.4]	[0.18, 0.61]
**PC SL/V**	---	[-1.89, -1.56]	[-1.87, -1.2]	[-2.11, -1.59]	[-2.1, -1.19]	[0.43, 0.86]

Table S5. Diffusion metrics for healthy controls. *NOTE: FA = fractional anisotropy; MD = mean diffusivity. Data displayed as mean ± standard deviation. MD in mm²/s.*

	Corpus Callosum		Corona Radiata		Centrum Semiovale	
	**FA**	**MD**	**FA**	**MD**	**FA**	**MD**
**HARDI**	0.65 ± 0.02	8.48E-04 ± 4.98E-05	0.45 ± 0.03	8.27E-04 ± 2.61E-04	0.51 ± 0.03	7.38E-04 ± 3.09E-05
**Sms**	0.69 ± 0.02	1.01E-03 ± 7.42E-05	0.50 ± 0.03	8.52E-04 ± 4.75E-05	0.50 ± 0.03	8.23E-04 ± 4.41E-05
**DSI**	0.69 ± 0.02	9.88E-04 ± 7.62E-05	0.50 ± 0.02	8.40E-04 ± 4.07E-05	0.50 ± 0.03	8.04E-04 ± 3.27E-05
**HCPms**	0.68 ± 0.02	9.88E-04 ± 6.26E-05	0.49 ± 0.02	8.35E-04 ± 3.74E-05	0.49 ± 0.03	8.01E-04 ± 3.82E-05

Table S6. Volumes (number of voxels) for the Sigmoid and Probst tracts in patients and Anterior and Posterior Commissures in healthy subjects. *NOTE: L-Probst Vol. = Left Probst Volume; R-Probst Vol. = Right Probst Volume; L-Sigmoid Vol. = Left Sigmoid Volume; R-Sigmoid Vol. = Right Sigmoid Volume; AC Vol. = Anterior Commissure Volume; PC Vol. = Posterior Commissure Volume; CC Vol. = Corpus Callosum Volume; L-CR Vol. = Left Corona Radiata Volume; R-CR Vol. = Right Corona Radiata Volume. Data displayed as mean ± standard deviation. Volumes are reported as the number of voxels in the tract-derived binary mask (voxels traversed by ≥1 streamline).*

	Patients				Controls				
	L-Probst Vol.	R-Probst Vol.	L-Sigmoid Vol.	R-Sigmoid Vol.	AC Vol.	PC Vol.	CC Vol.	L-CR Vol.	R-CR Vol.
**HARDI**	87580 ± 11644	88357 ± 11191	115387 ± 12634	118315 ± 11349	1141 ± 1015	1876 ± 1297	140497 ± 11866	50132 ± 6295	51300 ± 6358
**Sms**	39827 ± 8003	41675 ± 8347	25025 ± 9605	27061 ± 13843	2134 ± 2624	782 ± 1441	97409 ± 14235	30963 ± 5286	30020 ± 4721
**DSI**	45987 ± 7647	48329 ± 8952	36572 ± 7760	41151 ± 12297	3748 ± 3250	4826 ± 3995	106826 ± 10024	34909 ± 4073	34502 ± 4202
**HCPms**	46584 ± 7625	48190 ± 8640	35344 ± 7363	38501 ± 11657	2384 ± 1998	2751 ± 3410	107061 ± 10932	34217 ± 3419	33364 ± 3652
